# The first initiative of DNA barcoding of ornamental plants from Egypt and potential applications in horticulture industry

**DOI:** 10.1371/journal.pone.0172170

**Published:** 2017-02-15

**Authors:** Hosam O. Elansary, Muhammad Ashfaq, Hayssam M. Ali, Kowiyou Yessoufou

**Affiliations:** 1 Floriculture, Ornamental Horticulture and Garden Design Department, Faculty of Agriculture (El-Shatby), Alexandria University, Alexandria, Egypt; 2 Biodiversity Institute of Ontario, University of Guelph, ON, Guelph, Canada; 3 Botany and Microbiology Department, College of Science, King Saud University, Riyadh, Saudi Arabia; 4 Timber Trees Research Department, Sabahia Horticulture Research Station, Horticulture Research Institute, Agriculture Research Center, Alexandria, Egypt; 5 Department of Geography, Environmental Management and Energy Studies, University of Johannesburg, APK campus, Johannesburg, South Africa; Istituto di Biologia e Biotecnologia Agraria Consiglio Nazionale delle Ricerche, ITALY

## Abstract

DNA barcoding relies on short and standardized gene regions to identify species. The agricultural and horticultural applications of barcoding such as for marketplace regulation and copyright protection remain poorly explored. This study examines the effectiveness of the standard plant barcode markers (*matK* and *rbcL*) for the identification of plant species in private and public nurseries in northern Egypt. These two markers were sequenced from 225 specimens of 161 species and 62 plant families of horticultural importance. The sequence recovery was similar for *rbcL* (96.4%) and *matK* (84%), but the number of specimens assigned correctly to the respective genera and species was lower for *rbcL* (75% and 29%) than *matK* (85% and 40%). The combination of *rbcL* and *matK* brought the number of correct generic and species assignments to 83.4% and 40%, respectively. Individually, the efficiency of both markers varied among different plant families; for example, all palm specimens (Arecaceae) were correctly assigned to species while only one individual of Asteraceae was correctly assigned to species. Further, barcodes reliably assigned ornamental horticultural and medicinal plants correctly to genus while they showed a lower or no success in assigning these plants to species and cultivars. For future, we recommend the combination of a complementary barcode (e.g. *ITS* or *trnH*-*psbA*) with *rbcL* + *matK* to increase the performance of taxa identification. By aiding species identification of horticultural crops and ornamental palms, the analysis of the barcode regions will have large impact on horticultural industry.

## Introduction

Global horticultural industry is one of the fastest growing industries in agricultural sector. According to the US Bureau of Economic Analysis, floriculture related sales in the USA alone in 2012 were USD 27.8 billion while the sales for the global industry surpassed $60 billion. Unfortunately, the global market of horticultural industry is compromised by a wide range of counterfeited ornamental and fruit plants that have been sold without anyone paying intellectual propriety rights or following plant variety protection (PVP) laws [1,2]. Intellectual property infringements in horticultural crops may lead to large economic losses for plant breeders including small and medium size companies and public research institutes whose main revenues and license fees are paid by authorized producers, while illegal traders ignore the payment of such fees, and this results in negative impact not only on the producer but also on the society and global trade [3]. In the face of counterfeited ornamental plants and many other illegal activities in the industry, the development of reliable methods to distinguish among species or specimens of ornamental plants, fruits and vegetables may help in informing and enforcing the market regulations [4]. Traditionally, most plant identifications are based on morphological characters, but such identification is not always reliable and efficient [5]. A wide range of molecular techniques including (but not limited to) random amplified polymorphic DNA (RAPD) [6], amplified fragment length polymorphism (AFLP) [7], restriction fragment length polymorphism (RFLP) [8], microsatellite [9] and single nucleotide polymorphism (SNP) [10] have been proposed to identify plant species/specimen and cultivars. DNA barcoding has emerged as a relatively novel and perhaps more universal tool with which to analyze diversity of both plants and animals and to assign specimens to their species even in the absence of all or key morphological diagnostic features [11, 12]. Although there are still some reserves against the performance of DNA barcoding as compared, for example, to morphology, an early study, through a thorough comparison of DNA barcoding and morphology-based species identification recorded a number of limitations to the morphology particularly when it comes to cryptic species [5].

The earliest use of DNA barcoding to identify insect species [11] has triggered a global campaign that mobilizes scientists and institutions for biodiversity, ecology and phylogenetic studies [13–17]. The technique has become an acceptable taxonomic tool [18] and has been successfully used in large scale biodiversity projects where regional flora and fauna are documented [15,17,19], including regulated and threatened taxa [20]. Although a number of plant loci including, *trnH*-*psbA* [21], *rpoc1*, *rpoB* [22], *trnL* [23], *rbcL* [24] and *matK* [25] were initially proposed as potential plant barcodes based on assessments of recoverability, sequence quality and levels of species discrimination, the Consortium for the Barcode of Life [12] recommended the 2-loci combination of *rbcL* +*matK* as the standard plant barcode. However, there are persistent calls to include ITS into the core barcodes [26–28].

This combination *rbcL* +*matK* has been successful in several specimen identification campaigns across continents such as the barcoding campaign of the African rainforest trees in Cameroon [29], the trees and shrubs of Egypt [13], the forest trees in Panama [30], and the poorly known flora of Australia [31]. Similarly, several projects to barcode specific taxa such as the horticultural crops like *Ocimum* [32], *Ficus* [13,33], *Rhododendron* [34] and Araliaceae [35] have been initiated.

Specifically, some attention has been devoted to barcoding medicinal plants in China [36], and on the African continent it is only in South Africa that exceptional effort to DNA-barcode local and regional floras has been made [15,16,28]. However, in the northern Africa particularly in Egypt, a promising country for the production of ornamental crops [37] and a well-known country for its medicinal and horticultural plant diversity [38], the barcoding effort of local and regional floras is yet to be fueled although the applications of DNA barcoding a wide range of scientific disciplines are mounting; e.g. invasion ecology [4,39], biodiversity assessment [15], conservation efforts [16,40], and phylogeographic studies [40].

The present study is the first initiative of its kind in Egypt and northern Africa aiming to barcode the Egyptian ornamental herbs and palms in private and public nurseries. Specifically, we used the core DNA barcodes (rbc*L*a + *matK*) data that we generated to explore the resolution power of each marker in taxa and specimen identification. The DNA barcode data generated in the present study will serve in the future in commercial agricultural, horticultural and medicinal plant industries for the purpose of control of counterfeited product, and could also serve in ecological studies of local flora as demonstrated elsewhere [4,16,40].

## Materials and methods

### Plant material and tissue sampling

We collected 225 plant specimens from 161 taxa; of this collection, 121 specimens were sampled from the Green Oasis Nursery in Alexandria, 85 from the nursery of the Faculty of Science, Meharam Bek, Alexandria, 12 from Ashor Nursery in Montaza area in Alexandria, 5 from Mostafa Kamel Village Nursery in Mariout area in Alexandria and 2 from Antoniades garden in Alexandria (May 2016). All owners of the nurseries and gardens approved the sampling and publishing of the data and none of the plants were endangered or protected species. To examine the success of barcoding on ornamental plant cultivars several individuals belonging to the same species and differing in flower or leaf color were collected, and this included *Viola tricolor* (Hornveilchen lila, Frosthart, Hortensis, L., Heartsease, Hornveilchen hellgelb, Simon Shine, Sun Glory, Freefall Purple & White) and *Pericallis x hybrida* (Senetti Blue Bicolor, Senetti Magenta, Senetti Super Blue, Senetti Pink, Jester Pure White) etc.. Samples collections, analyses and vouchering were completed in May 2016. These specimens were geo-referenced with digital pictures and leaf samples were dried in silica gel for subsequent analysis. Specimen information along with images is available on Egypt barcode of life project (www.boldystems.org) and S1 Table.

### DNA extraction, PCR and sequencing

DNA extraction, PCR amplification and sequencing were performed at the Canadian Centre for DNA Barcoding (CCDB) of the Biodiversity Institute of Ontario. DNA extractions, PCR amplifications and sequencing were performed following CCDB protocols (S1 and S2 Sheets). The following primers were used for amplification and sequencing: *rbcL*: *rbcL*-F (TGTCACCACAAACAGAGACTAAAGC) [41], *rbcL*-R(GTAAAATCAAGTCCACCRCG) and *matK*: *MatK*-1RKIM-f (CCCAGTCCATCTGGAAATCTTGGTTC), *MatK*-3FKIM-r (GTACAGTACTTTTGTGTTTACGAG). The forward and reverse trace files were trimmed and assembled after sequencing using the CodonCode Aligner V 3.5.4 (CodonCode Co., USA). All the sequences generated are available on Genbank/EBI (*matK* accession No. KX783623—KX783811; *rbcL* accession No. KX783812-KX784028).

### Statistical analysis

BLAST tests against the GenBank database were performed for identification of specimens at family, genus and species levels and the resolution efficiency was determined based on Blast1 method (BLAST1: the ID is that of the species associated with the best BLAST hit, and E-value<cut-off. This corresponds to choosing the top hit in the BLAST results) [42]. The correct identification means that the individual is assigned to the right species, genus or family; ambiguous identification means that the individual is assigned to one or several species, genera or families including the right one; incorrect identification means that the individual is assigned to one or several species, genera or families not including the right one [43]. TAXONDNA [44] was used to assess the distribution of interspecific and intraspecific distances in the dataset. Barcode gap analysis of *matK* and *rbcL* was performed using Kimura 2-parameter distance model implemented in Boldsystems [45]. Consensus barcode of each species was obtained using the ‘Consensus Barcode Generator’ function of TAXONDNA [44]. Consensus barcodes were used in a neighbor-joining (NJ) trees of *matK*, *rbcL* and the combined *rbcL* + *matK* sequences using evolutionary distances computed based on the Kimura 2-parameter [46] method in MEGA6 [47]. Sequences were trimmed, and aligned using MUSCLE [48] by pairwise deletion and 500 replications of Bootstrap phylogeny test. Distance analyses were performed in MEGA6 between families, within families and among species using consensus barcode sequences. The number of segregation sites and nucleotide diversity value which is the average number of nucleotide differences per site between a pair of randomly chosen sequences [49] was calculated for *matK* and *rbcL* using DnaSP v5 [50]. All alignments are available as S1–S3 Alignments.

## Results

### Sequencing success

PCR amplifications of 225 plant specimens yielded 217 (96.4%) *rbcL* and 189 (84%) *matK* sequences. Our collection represented 161 plant species, 98.1% of them were successfully sequenced for *rbcL* and 83.9% for *matK*. Sequence length distribution ranged between 506-552bp and 468–894 bp for *rbcL* and *matK*, respectively. The longest *matK* sequences (894 bp) were produced for *Ipomoea*, *Mentha and Syngonium* while the shortest (468 bp) in *Mattiola incana* (L.) R.Br. For *rbcL* most species produced similar length (552 bp) except for few short fragments in *Bauhinia retusa* (520 bp), *Papaver rhoeas* L. (531 bp), *Spiraea cantoniensis* (529 bp) and in *Rosa hybrida* L. (215 bp). The GC% ranged from 27.98 to 83.34 with an average of 33.64 in *matK* whereas in *rbcL* it ranged from 40.29 to 43.30 with an average of 36.38. Mean number of specimens examined per species was 1.44 and 1.45 for *rbcL* and *matK*; respectively. Sequencing success varied between families (S2 Table). The lowest success rates were found in *matK* in several members of Crassulaceae (12.5%), Malvaceae (57.14%) and Brassicaceae (66.7%). Furthermore, some singleton families (represented by one member) were not amplified or sequenced in *matK* such as Balsaminaceae, Oxalidaceae and others. The *rbcL* showed 100% amplification and sequencing success with most families except for few members of the family Linaceae, Piperaceae, Araceae, Lamiaceae, and Asteraceae. Medicinal and horticultural families such as Lamiaceae showed high sequences recoveries (100 *matK*, 91.3% rbcL).

### Species resolution and barcode analyses

Using *matK* sequences, taxa were correctly assigned by 100, 85.2 and 39.7% at the family, genus and species levels, respectively, whereas ambiguous identification was 6.9 and 36.5% for genus and species levels. Incorrect *matK* identification represented 7.9 and 23.8% for the genus and species, respectively (Table 1). The *rbcL* successfully identified 100, 74.65 and 29% at the levels of family, genus and species, respectively, whereas ambiguous identification was 13.8 and 38.2% for genus and species levels. Incorrect *rbcL* identification represented 11.5 and 32.7% for genus and species, respectively. Concatenations of *matK* & *rbcL* sequences correctly assign 83.4% taxa to their genus and 39.8% to species while it assigned 11.6% of genera and 46.9% of species ambiguously. By concatenating rbcL and matK, the incorrect assignments were only 4.9% for the genera and 13.3% for species.

**Table 1:** Identification success of Egyptian horticultural crops based on Blast1 method.

		Correct (%)	Ambiguously identified (%)	Incorrect identification (%)	Total sample Number	Average sequence Length bp
***Species***	*rbcL*	63 (29.03)	83(38.24)	71 (32.72)	217	522
	*matK*	75 (39.68)	69(36.50)	45 (23.80)	189	870
	*rbcLa+matK*	72 (39.77)	85(46.96)	24(13.25)	181	1323
***Genus***	*rbcL*	162 (74.65)	30 (13.82)	25 (11.52)	217	522
	*matK*	161 (85.18)	13(6.87)	15 (7.90)	189	870
	*rbcLa+matK*	151(83.42)	21(11.60)	9(4.90)	181	1323
***Family***	*rbcL*	217 (100)	-	-	217	522
	*matK*	189 (100)	-	-	189	870
	*rbcLa+matK*	181(100)	-	-	181	1323

In TaxonDNA, pairwise intraspecific distances in the two barcode loci of all dataset ranged from 0.0–2.7% (Table 2). The *rbcl+matK* showed higher mean intraspecific value than either marker. Pairwise mean interspecific distances were low (0.4%) in *rbcL* and high (1.3%) in *matK*. The concatenation of barcode loci did not increase the interspecific mean distances (Table 2). The data showed overlapping between intraspecific and interspecific distances of the individual or combined sequences (Table 2). This overlapping did not differ between *rbcL* (89.3%) and *matK* (89.4%) while being increased in *rbcl+matK* (97.2%). The barcode gap analysis provides the distribution of distances within each species and the distance to the nearest neighbor (NN) of each species. The use of barcode gap analysis tool on BOLD for *matK* under K2P distance model (pairwise deletion) showed a higher mean NN distances (4.7) than the mean intraspecific (0.01) indicating the existence of a barcode gap. Based on 189 *matK* sequences 22 species showed a higher (>2%) and 52 showed a lower (<2%) intraspecific divergence. The *rbcL* showed a higher mean (2.3) NN than the mean intraspecific (0.0) distance. The analysis of 217 *rbcL* sequences showed 23 species with higher (>2%) and 91 with lower (<2%) intraspecific distances.

**Table 2:** Pairwise intraspecific and interspecific distances in the barcode loci of all 161 plant species.

	Intraspecific distances (%)			Interspecific distances (%)			Intra-/interspecific distance overlap with 5% error margin on both sides	
**Locus (n)**	Min	Max	Mean	Min	Max	Mean	Overlapping distance range (%)	Intra-/interspecific sequences in the overlap (%)
***matK* (189)**	0.00	0.15	0.02	0.00	11.35	1.28	0.0–0.24	89.41
***rbcL*(217)**	0.00	0.18	0.01	0.00	3.08	0.43	0.0 to 0.18	89.33
***matK+rbcL* (182)**	0.00	2.71	0.24	0.00	8.31	1.17	0.0 to 3.15	97.17

### Sequence analysis

#### Families and genera clustering

The NJ tree for *rbcL*+*matK* was generated using 182 sequences (S1 Fig) with at least one sequence from each family. Members of each family are clustered on the tree with the largest cluster for the family Lamiaceae in the *matK* (S1 Fig) or *rbcL* + *matK* trees. Furthermore, each genus was split into sub-clusters. In Solanaceae, 13 individuals from 6 genera were clustered. Barcodes separated all the genera but did not separate a majority of the species. Members of Asparagaceae were analyzed by both markers and formed two subclusters, one joined the genera *Yucca* and *Chlorophytum* and the second joined *Dracaena*, *Sansveiria*, *Asparagus* and *Cordyline* (S1 Fig). In Arecaceae, nine species were examined. Species of Arecaceae were differentiated in *matK* and *rbcL+matK* but formed one cluster. The taxa *Spathiphyllum*, *Monstera*, *Anthurium*, *Aglonema* were discriminated by both loci except for three species belonging to *Phillodendron*.

#### Simple diagnostic characters for genera and species

**Fig 1:**
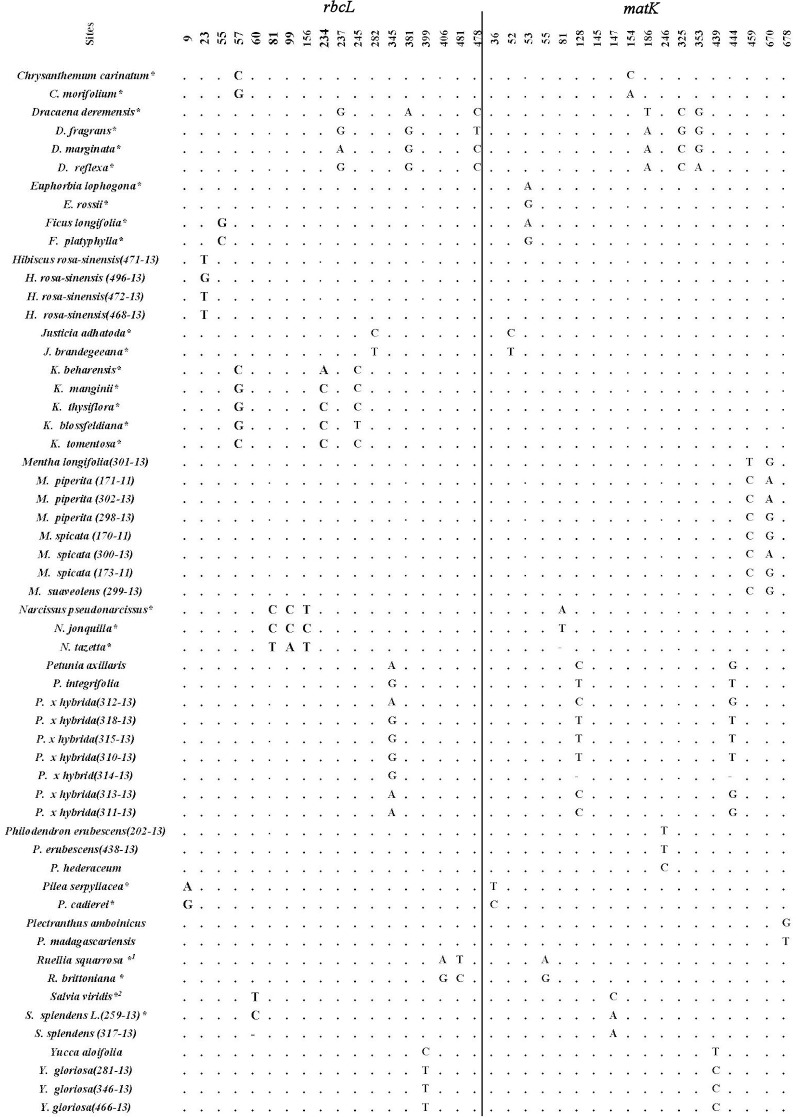
Within genera polymorphic sites identified for *rbcL* and *matK*. (*) indicating other polymorphic sites not shown in both markers. (1) indicating 111-6bp deletion, (2) indicating 117-6bp deletion.

*Mentha* showed simple diagnostic characters as two polymorphic sites in the local species split the genus into three different haplotypes (Fig 1). The first contained 459-T&670-G, exclusively found in *M*. *longifolia* L. whereas the second and third haplotypes (459-C &670-A /459-C &670-G) were shared in *M*. *piperita* L., *M*. *suaveolens* Apple mint *and M*. *spicata* L. Two haplotypes of *matK* were *found* in *Plectranthus* (Lamiaceae); one of them had 678-T (*P*. *madagascariensis var*.madagascariensis) and the second 678-G (*P*. *amboinicus* "spanish thyme") associated with morphological variation such as leaves variegation in the former. In *Salvia* (Lamiaceae), two haplotypes were found in each locus; one of them is associated with *S*. *viridis* L. and the second in two *S*. *splendens* Sellow ex Schult. Two species in Lamiaceae [*Rosmarinus officinallis* L., *Solenostemon scutellarioides* (different cultivars)] did not show diagnostic characters although the former shows clear morphological differences among subspecies examined. In *Petunia* (Solanaceae), 3 species were examined (*P*.*x hybrida*, *P*. *axillaris* and *P*. *integrifolia*), two haplotypes in each of *matK* and *rbcL* were found. Each of *Petunia axillaris* (Lam.) Britton, Sterns & Poggenb. and *P*. *integrifolia* subsp. *inflata* had its own haplotype in each barcode marker whereas *P*. *x hybrida* cultivars contained both haplotypes of each barcode and each barcode marker divided *P*. *x hybrida* cultivars into two groups based on single nucleotide polymorphism. In *Dracaena* (Asparagaceae) four morphologically divergent species were barcoded and each barcode differentiated each species accurately where four haplotypes were produced in each locus. Furthermore, other species of Asparagaceae such as *Yucca gloriosa* variegata and *Y*. *aloifolia* purpurea produced two haplotypes in both loci. In Arecaceae, species of the genera *Dypsis*, *Livistona*, *Ravenea* and *cocos* showed clear diagnostic characters. *Monstera*, *Spathiphyllum*, *Anthurium*, *Aglonema*, *Zamiocolocas* (Araceae) had their own simple diagnostic characters in both markers. Simple diagnostic characters were found in the closely related genera of *Chrysanthemum* and *Matricaria* of the family Asteraceae.

#### Genetic distances among families, species and nucleotide diversity

We compared maximum, minimum and average distances for each locus and for the combined *rbcL+matK* sequence. In *matK*, mean distance among families, within families and among species were 0.22, 0.05 and 0.22; respectively (Table 3). In *rbcL* mean distance among families, within families and among species were 0.09, 0.02 and 0.09; respectively. *rbcL+matK* showed the largest distances compared to individual locus. However, minimum distances among families in individual or combined loci were higher than minimum within families or among species.

**Table 3:** Genetic distances in barcode loci at three taxonomic levels.

	Among families			within families			species		
**Barcode locus (n = 132)**	min	Max	Mean	Min	Max	Mean	Min	Max	Mean
***matK***	0.05	0.38	0.22	0	0.12	0.05	0	0.4	0.22
***rbcL***	0.02	0.16	0.09	0	0.06	0.02	0	0.17	0.09
***rbcL+matK***	0.04	0.14	0.24	0	0.08	0.034	0	0.24	0.14

**Fig 2:**
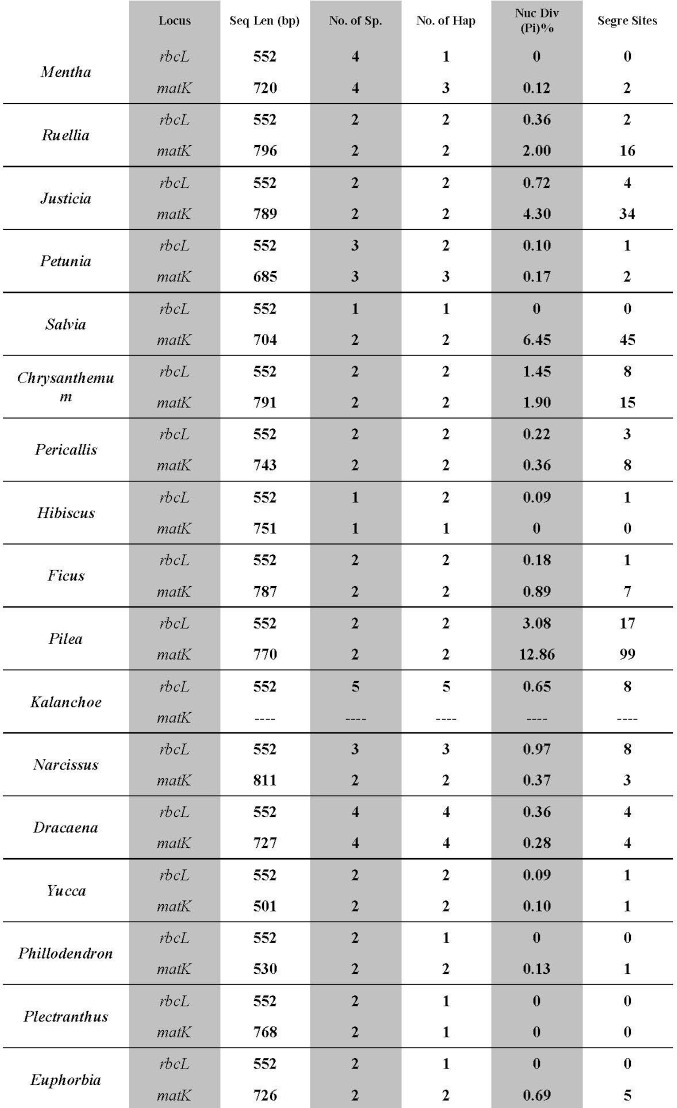
Comparison of two loci tested on several genera. No.: Number, Sp.: Species, Seq.: Sequence, Len.: Length, Hap.: Haplotype, Nuc. Div.: Diversity, Segre.: Segregation.

Nucleotide diversity, number of segregation sites and number of haplotypes for the two barcode loci for all genera represented by several species were calculated (Fig 2). The number of species ranged from 1 (*Hibiscus rosa-sinensis* L.) in *matK* and *rbcL* to 5 in *rbcL* such as in the genus *Kalanchoe* (*K*. *beharensis* Drake, *K*. *blossfeldiana* Poelln., K. *manginii* Raym.-Hamet & H.Perrier, *K*. *thysiflora* Balfour and *K*. *tomentosa* Golden Girl) as shown in Fig 2 and in S1 Table. The highest number of segregation sites in all genera was in *Pilea* between *P*. *cadierei* Gagnep. & Guillaumin and *P*. *serpyllacea* (Kunth) Liebm. The *Pilea* was followed by *Salvia* cultivars and *Justicia* cultivars for *matK*. The nucleotide diversity ratio followed the same trend as for the segregation sites where the highest value was in the *Pilea* followed by *Salvia* (*S*. *splendens* Sellow ex Schult. & *S*. *virdis* L.) and *Justicia* (J. adhatoda L. & *J*. *brandegeeana* Wassh. & L.B.Sm.) in both loci. In *matK*, the number of haplotypes ranged from 1–4 (Fig 2). Nucleotide diversity was highest in *Pilea* (12.9%) followed by *Salvia* (6.5%) and *Justicia* (4.3%) whereas the remaining genera ranged from 2–0%. In *rbcL*, the number of haplotypes was either high (5, 4 and 3) such as in *Kalanchoe*, *Dracaena* and *Narcissus* L., respectively or low (2 or 1) in all remaining genera. The highest values were found in *Pilea* (3.08), *Chrysanthemum* L. (1.45), *Narcissus* (0.97), *Justicia* (0.72) and *Dracaena* (0.36).

## Discussion

DNA barcoding campaign is still at its infancy stage in Africa particularly in northern Africa, although an increasing effort is noted in South Africa [4,15,16]. Our study, a first attempt of DNA barcoding study of its kind in Egypt and northern Africa, showed a higher sequencing success for *rbcL* than *matK*. Previous studies have shown a similar pattern in other plant groups [24,13]. Our sequencing success of *matK*, however, matched that reported in CBOL [12] but was higher than that reported by Parmentire et al. [29]. The sequences recovery in the family Lamiaceae was higher in *matK* than *rbcL* disagreeing with a study by Theodoridis et al. [51] on the same plant family. We found a higher universality in *rbcL* in genera identification but a lower species resolution than what was observed in *matK* [12]. In addition, we found a barcode gap [52] in *matK* with a higher mean interspecific than the mean intraspecific distances in 189 sequences. In general, the barcode gaps observed in this study is higher than that found in an early study of trees and shrubs in Egypt [13]. Although the existence of barcode gap may not predict the discrimination success [53], it is a key criterion for barcoding assessment. Genetic distance analyses were conducted at different taxonomic levels. Distances with *matK* were 2 times greater than the mean distance of *rbcL* in all cases, indicating a higher resolution power of matK for the poorly studied flora of Egypt. Furthermore, concatenated sequences of *rbcL* and *matK* slightly increased distances reflecting improved resolution power using both barcodes which is in agreement with Parmentier et al. [29] and Saarela et al. [54]. Both barcodes indicated that the largest genetic distance was achieved within Rubiaceae between *Pentas lancedata* (Forssk.) Deflers and *Hoffmania discolor* (Lem.) Hemsl. The family Rubiaceae contains over 13,200 species in 620 genera in addition to numerous unresolved generic complexes [55] and the family harbors a high diversity, especially in southern African countries and South America and has a worldwide distribution. The high number of segregation sites and consequently high nucleotide diversity found in *Pilea* (Urticaceae) species compared to other genera is due to the species richness of this genus as it contains over 700 species and one fifth of the diversity of seed plants [56,57].

As expected, taxonomic assignment decreases from family, genus to specific level [29,13]. A combination of *rbcL*+*matK* slightly improved the rate of correct species resolution over the individual markers. The combined markers did not improve genus identification, supporting previous report for African flora [29]. The combined markers, however, dramatically reduced the level of incorrect species identification by 60% in *rbcL* and 44% in *matK*. A similar trend was found at the genus level. Correct cultivar assignments were 1.4 and 1.3% for *matK* and *rbcL*, respectively. The lower species discrimination in our study could be attributed to several factors such as floristic affinities (e.g. close relatives are well known for not being easily discriminated by the official barcodes [58]), or the existence of multiple cultivars in our horticultural crops. Further, it is also possible, owing to the Egyptian flora being understudied, that there is a taxonomic confusion (vague morphological parameters leading to misidentifications) in the existing morphology-based species discrimination.

We compared our *matK* sequences from mint species with those in GenBank and constructed a phylogenetic tree (data not shown). The identification rate was low; one potential reason could be possible hybridization, introgression or gene flow between species [59] blurring both genetic and taxonomic delimitations between taxa [60]. It could also be because the GenBank data are questionable as such doubt about public repositories has previously been reported (e.g. [61]). It is also likely that the well-known maternal inheritance associated with plastid regions [62] plays a role in the poor discriminatory power of *rbcL* and matK. Furthermore, although we expect nuclear region that could perform better than *rbcL* + *matK*, several recent studies also reported low performance (i.e. ≤ 50%) for *ITS*. For example, the highest performance of *ITS* for Orchids was around 50% [63] and about 30% for Alooidea [28]. Our objective in this study was to build a DNA barcode library for Egyptian flora and demonstrate how DNA barcodes data can be used for biodiversity assessment, and ecological studies of local flora in future studies (e.g. see ref. [16,39] for South Africa’s flora).

Although identification rate is known to decrease with an increase in the mean number of species per genus [29], this could not be the case in this study as the mean number of species per genus is lower (1.3) than reported in other studies [64]. Low identification rate in both core barcodes is common as reported in several taxonomic groups: Indian Berberis (23%; [65]), Pinaceae (25%; [66] and vascular plants of Manitoba, Canada (45–55%; [67] and African Combretaceae (10–61%; [15]). Dong et al. [68] explored the use of *rbcL* as barcode in all plant families and found that the successful species identification rates varied significantly among plant groups, ranging from 24.58% to 85.50%.

Furthermore, NJ-tree analysis shows that both Asparagaceae and Amarillidaceae are sisters in the tree (S1 Fig) which is in agreement with the Angiosperm phylogeny Group (APGIII) tree (http://www.mobot.org/MOBOT/Research/APweb/welcome.html). *matK* highly discriminated species of Arecaceae, suggesting that *matK* is a strong DNA barcode candidate for the Egyptian palms. In addition, 13 species demonstrated simple diagnostic characters whereas other species had homologous sequences using both core barcodes. Sequence variation in some cases was associated with morphological variations and in other cases sequences were identical. Our study therefore recommends the use of several combined markers beyond *rbcL* and *matK*. The two species of *Slavia* (*S*. *splendens* Sellow ex Schult. & *S*. *virdis* L.) examined showed simple diagnostic characters in both markers, matching the morphological difference between both species based on the flower color (red in *S*. *splendens* and blue in *S*. *viridis*). Barcodes discriminated between the two closely genera of *Chrysanthemum* (*C*. *carinatum* Schousb., *C*. *morifolium* Ramat.) and *Matricaria* (*M*. *chamomilla* L.) of the family Asteraceae. Morphologically divergent varieties or hardly known varieties were chosen from these genera to be barcoded in this study. In some cases, we chose varieties showing variation in flowers color such as in *Viola tricolor* (Hornveilchen lila, Frosthart, Hortensis, L., Heartsease, Hornveilchen hellgelb, Simon Shine, Sun Glory, Freefall Purple & White), *Pericallis x hybrida* (Senetti Blue Bicolor, Senetti Magenta, Senetti Super Blue, Senetti Pink, Jester Pure White) and *Antirrhinum majus* L. (pink and white). In other cases, we studied varieties showing variation in leaf-shape and variegation in leaf color such as in *Brassica olearaceae* (Emperor white, L., Dietrich Idaho and Nagoya Red F1), *Hydrangea macrophylla* (Thunb. Ser. and L.) and *Codiaeum variegatum* (L.) Rumph. ex A. Juss. Only four of all species examined either for *matK* or in *rbcL* showed variation among varieties in the ratio of 1.4% and 1.4%; respectively. The inability to distinguish among subspecies/varieties using the core barcodes is well established [29] although few cases where barcodes or plastid regions were successful in discriminating among subspecies as found in *Mentha spicata* L. and *M*. *x piperita* (Chocolate and L.) and the case of the intergenic spacer *trnH-psbA* (a complementary DNA barcode) in *Silene vulgaris* (Moench) Garcke [69] as well as *matK* and *rbcL* in *Celtis occidentalis* L. [13] were also reported.

## Conclusion

The application of DNA barcoding in horticultural and agricultural industry is promising. Both the core barcodes have a high resolution power at genus level and moderate at the species level with *matK* showing higher resolution power at all taxonomic levels. The addition of other barcodes may enhance the discriminatory power of barcoding at genus and species levels. The core DNA barcodes are not always able to discriminate species but have more promise in controlling the market place of horticultural crops and protecting copyrights of new species or cultivars. Nuclear markers are generally advocated for, and the ITS region in particular, although we should acknowledge some controversies around this nuclear marker (see [70]): incomplete lineage sorting, inhomogeneous concerted evolution, divergent paralogous copies within individuals, and pseudogenes; [71]; but see ref. [26]). Overall, we suggest that including more replicates per species and adopt a more multi-gene approach that includes a nuclear region may result in a more efficient DNA barcode data for horticultural and agricultural industry.

## Supporting information

S1 Table Taxa included in the study. (DOCX)

S2 Table Families using each of matK & rbcLa of BOLD Taxon ID tree analysis showing that there are 189 sequences, 117 species, 114 genus and 50 family using matK and 217 sequences, 131 species, 132 genus and 62 family using rbcLa. % of PCR success based on families is also illustrated.
(DOCX)

S1 Alignment *rbcL* alignment. (FAS)

S2 Alignment *matK* alignment. (FAS)

S3 Alignment *rbcL+matK* alignment. (FAS)

S1 Fig NJ tree of *rbcL* and *matK* produced in MEGA6. (DOCX)

S2 Fig NJ tree of taxa using *matK*, produced in MEGA 6. (DOCX)

S3 Fig NJ tree of *rbcL* produced in MEGA6. (DOCX)

S1 Sheet CCDB DNA extraction for plants. (PDF)

S2 Sheet CCDB PCR amplification for plants. (PDF)
