# Supplementary material for: The first initiative of DNA barcoding of ornamental plants from Egypt and potential applications in horticulture industry
Source: PLoS One. 2017 Feb 15;12(2):e0172170. doi: 10.1371/journal.pone.0172170 (PMC5310869; doi:10.1371/journal.pone.0172170)
Supplement: S1 Table — (DOCX) [file pone.0172170.s001.docx]

S1 Table. Taxa included in the study.

| No. | Identification | Race/Variety | Family | Vouchering Data  Museum ID/Sample ID | GenBank Aaccession No. | |
| --- | --- | --- | --- | --- | --- | --- |
|  |  |  |  |  | *matK* | *rbcL* |
| 1 | *Abutilon darwinii* | Hook. f. | Malvaceae | HOSAM233-13 | KX783623 | KX783812 |
| 2 | *Acokanthera spectabilis* | (Sond.) Hook.f. | Apocynaceae | HOSAM364-13 | KX783624 | KX783813 |
| 3 | *Aerva sanguinolenta* | (L.) Blume | Amaranthaceae | HOSAM367-13 | KX783625 | KX783814 |
| 4 | *Afrocarpus gracilior* | (Pilg.) C.N.Page | Podocarpaceae | HOSAM243-13 | - | KX783815 |
| 5 | *Agave amricana* | L. | Asparagaceae | HOSAM071-10 | - | KX783816 |
| 6 | *Aglaonema commutatum* | Schott | Araceae | HOSAM256-13 | KX783626 | KX783817 |
| 7 | *Ajuga reptans* | L. | Lamiaceae | HOSAM434-13 | KX783627 | KX783818 |
| 8 | *Alternanthera dentate* | Little Ruby | Amaranthaceae | HOSAM433-13 | KX783628 | KX783819 |
| 9 | *Ananas comosus* | Variegatus | Bromeliaceae | HOSAM432-13 | - | KX783820 |
| 10 | *Anethum graveolens* | L. | Apiaceae | HOSAM287-13 | KX783629 | KX783821 |
| 11 | *Anthurium acaule* | (Jacq.) Schott | Araceae | HOSAM426-13 | KX783630 | KX783822 |
| 12 | *Antigonon leptopus* | Hook. & Arn. | Polygonaceae | HOSAM222-13 | KX783631 | KX783823 |
| 13 | *Antirrhinum majus* | L. | Plantaginaceae | HOSAM263-13 | KX783632 | KX783824 |
| 14 | *Antirrhinum majus* | L. | Plantaginaceae | HOSAM264-13 | KX783633 | KX783825 |
| 15 | *Aphelandra squarrosa* | Nees | Acanthaceae | HOSAM250-13 | KX783634 | KX783826 |
| 16 | *Areca catechu* | L. | Arecaceae | HOSAM476-13 | KX783635 | KX783827 |
| 17 | *Argyreia nervosa* | (Burm. f.) Bojer | Convolvulaceae | HOSAM204-13 | KX783636 | KX783828 |
| 18 | *Aristolochia littoralis* | Parodi | Aristolochiaceae | HOSAM216-13 | - | KX783829 |
| 19 | *Artemisia abrotanum* | L. | Asteraceae | HOSAM366-13 | KX783637 | KX783830 |
| 20 | *Asparagus plumosus* | Baker | Asparagaceae | HOSAM293-13 | KX783638 | KX783831 |
| 21 | *Asplenium antiquum* | Makino | Aspleniaceae | HOSAM425-13 | - | KX783832 |
| 22 | *Aucuba japonica* | Variegata | Garryaceae | HOSAM428-13 | KX783639 | KX783833 |
| 23 | *Bauhinia retusa* | Poir. | Fabaceae | HOSAM242-13 | KX783640 | KX783834 |
| 24 | *Begonia semperflorens* | Hook. | Begoniaceae | HOSAM246-13 | KX783641 | KX783835 |
| 25 | *Brassica oleracea* | Emperor white | Brassicaceae | HOSAM341-13 | - | KX783836 |
| 26 | *Brassica oleracea* | L. | Brassicaceae | HOSAM194-13 | - | KX783837 |
| 27 | *Brassica oleracea* | Dietrich Idaho | Brassicaceae | HOSAM340-13 | KX783642 | KX783838 |
| 28 | *Brassica oleracea* | Nagoya Red F1 | Brassicaceae | HOSAM339-13 | KX783643 | KX783839 |
| 29 | *Bryophyllum tubiflora* | L. | Crassulaceae | HOSAM282-13 | - | KX783840 |
| 30 | *Calendula officinalis* | L. | Asteraceae | HOSAM218-13 | KX783644 | KX783841 |
| 31 | *Caryota mitis* | Lour. | Arecaceae | HOSAM350-13 | KX783645 | KX783842 |
| 32 | *Cestrum nocturnum* | L. | Solanaceae | HOSAM286-13 | KX783646 | KX783843 |
| 33 | *Chasmanthe aethiopica* | (L.) N.E.Br. | Iridaceae | HOSAM230-13 | KX783647 | KX783844 |
| 34 | *Chlorophytum comosum* | (Thunb.) Jacques | Asparagaceae | HOSAM392-13 | KX783648 | KX783845 |
| 35 | *Chlorophytum comosum* | (Thunb.) Jacques | Asparagaceae | HOSAM403-13 | KX783649 | KX783846 |
| 36 | *Chrysanthemum carinatum* | Schousb. | Asteraceae | HOSAM260-13 | KX783650 | KX783847 |
| 37 | *Chrysanthemum morifolium* | Ramat. | Asteraceae | HOSAM280-13 | KX783651 | KX783848 |
| 38 | *Clerodendron splendens* | G.Don | Lamiaceae | HOSAM234-13 | KX783652 | KX783849 |
| 39 | *Cocos nucifera* | L. | Arecaceae | HOSAM473-13 | KX783653 | KX783850 |
| 40 | *Codiaeum variegatum* | (L.) Rumph. ex A.Juss. | Euphorbiaceae | HOSAM347-13 | KX783654 | KX783851 |
| 41 | *Codiaeum variegatum* | (L.) Rumph. ex A.Juss. | Euphorbiaceae | HOSAM270-13 | - | KX783852 |
| 42 | *Codiaeum variegatum* | (L.) Rumph. ex A.Juss. | Euphorbiaceae | HOSAM271-13 | KX783655 | KX783853 |
| 43 | *Codiaeum variegatum* | (L.) Rumph. ex A.Juss. | Euphorbiaceae | HOSAM267-13 | KX783656 | - |
| 44 | *Codiaeum variegatum* | (L.) Rumph. ex A.Juss. | Euphorbiaceae | HOSAM266-13 | KX783657 | KX783854 |
| 45 | *Codiaeum variegatum* | (L.) Rumph. ex A.Juss. | Euphorbiaceae | HOSAM268-13 | KX783658 | KX783855 |
| 46 | *Codiaeum variegatum* | (L.) Rumph. ex A.Juss. | Euphorbiaceae | HOSAM348-13 | KX783659 | KX783856 |
| 47 | *Codiaeum variegatum* | (L.) Rumph. ex A.Juss. | Euphorbiaceae | HOSAM215-13 | KX783660 | KX783857 |
| 48 | *Cordyline australis* | Red Star | Asparagaceae | HOSAM394-13 | KX783661 | KX783858 |
| 49 | *Crassula rupestris* | L.f. | Crassulaceae | HOSAM402-13 | - | KX783859 |
| 50 | *Crinum longifolium* | L. | Amaryllidaceae | HOSAM228-13 | KX783662 | KX783860 |
| 51 | *Cyperus papyrus* | L. | Cyperaceae | HOSAM206-13 | KX783663 | KX783861 |
| 52 | *Datura stramonium* | L. | Solanaceae | HOSAM225-13 | KX783664 | KX783862 |
| 53 | *Dodonaea viscosa* | (L.) Jacq. | Sapindaceae | HOSAM273-13 | KX783665 | KX783863 |
| 54 | *Dombeya rotundifolia* | (Hochst.) Planch. | Malvaceae | HOSAM231-13 | - | KX783864 |
| 55 | *Dracaena deremensis* | var. warneckei Engl. | Asparagaceae | HOSAM443-13 | KX783666 | KX783865 |
| 56 | *Dracaena fragrans* | (L.) Ker Gawl. | Asparagaceae | HOSAM258-13 | KX783667 | KX783866 |
| 57 | *Dracaena marginata* | hort. | Asparagaceae | HOSAM203-13 | KX783668 | KX783867 |
| 58 | *Dracaena reflexa* | Lam. | Asparagaceae | HOSAM441-13 | KX783669 | KX783868 |
| 59 | *Duranta erecta* | L. | Verbenaceae | HOSAM419-13 | KX783670 | KX783869 |
| 60 | *Dypsis lutescens* | (H.Wendl.) Beentje & J.Dransf. | Arecaceae | HOSAM446-13 | KX783671 | KX783870 |
| 61 | *Dypsis lutescens* | (H.Wendl.) Beentje & J.Dransf. | Arecaceae | HOSAM475-13 | KX783672 | KX783871 |
| 62 | *Dypsis lutescens* | (H.Wendl.) Beentje & J.Dransf. | Arecaceae | HOSAM467-13 | KX783673 | KX783872 |
| 63 | *Erysimum cheiri* | (L.) Crantz | Brassicaceae | HOSAM195-13 | KX783674 | KX783873 |
| 64 | *Euonymus japonicus* | Thunb. | Celastraceae | HOSAM465-13 | KX783675 | KX783874 |
| 65 | *Euonymus japonicus* | Thunb. | Celastraceae | HOSAM464-13 | KX783676 | KX783875 |
| 66 | *Euphorbia lophogona* | Lam. | Euphorbiaceae | HOSAM456-13 | KX783677 | KX783876 |
| 67 | *Euphorbia rossii* | Rauh & Buchloh | Euphorbiaceae | HOSAM457-13 | KX783678 | KX783877 |
| 68 | *Fatsia japonica* | (Thunb.) Decne. & Planch. | Araliaceae | HOSAM429-13 | KX783679 | KX783878 |
| 69 | *Ficus longifolia* | Schott | Moraceae | HOSAM410-13 | KX783680 | KX783879 |
| 70 | *Ficus platyphylla* | Delile | Moraceae | HOSAM217-13 | KX783681 | KX783880 |
| 71 | *Freesia alba* | (G.L.Mey.) Gumbl. | Iridaceae | HOSAM290-13 | KX783682 | KX783881 |
| 72 | *Freesia refracta* | (Jacq.) Klatt | Iridaceae | HOSAM291-13 | KX783683 | KX783882 |
| 73 | *Gardenia augusta* | Merr. | Rubiaceae | HOSAM396-13 | KX783684 | KX783883 |
| 74 | *Godetia whitneyi* | (A.Gray) T.Moore | Onagraceae | HOSAM284-13 | KX783685 | KX783884 |
| 75 | *Guzmania lingulata* | (L.) Mez | Bromeliaceae | HOSAM387-13 | - | KX783885 |
| 76 | *Hibiscus rosa-sinensis* | L. | Malvaceae | HOSAM469-13 | KX783686 | KX783886 |
| 77 | *Hibiscus rosa-sinensis* | L. | Malvaceae | HOSAM472-13 | KX783687 | KX783887 |
| 78 | *Hibiscus rosa-sinensis* | L. | Malvaceae | HOSAM468-13 | - | KX783888 |
| 79 | *Hibiscus rosa-sinensis* | L. | Malvaceae | HOSAM471-13 | - | KX783889 |
| 80 | *Hoffmannia discolor* | (Lem.) Hemsl. | Rubiaceae | HOSAM236-13 | KX783688 | KX783890 |
| 81 | *Hydrangea macrophylla* | (Thunb.) Ser. | Hydrangeaceae | HOSAM420-13 | KX783689 | KX783891 |
| 82 | *Hydrangea macrophylla* | L. | Hydrangeaceae | HOSAM427-13 | KX783690 | KX783892 |
| 83 | *Hymenocallis caribaea* | (L.) Herb. | Amaryllidaceae | HOSAM451-13 | KX783691 | KX783893 |
| 84 | *Hypoestes phyllostachya* | Baker | Acanthaceae | HOSAM412-13 | KX783692 | KX783894 |
| 85 | *Hypoestes phyllostachya* | Baker | Acanthaceae | HOSAM413-13 | KX783693 | KX783895 |
| 86 | *Hypoestes phyllostachya* | Baker | Acanthaceae | HOSAM414-13 | KX783694 | KX783896 |
| 87 | *Impatiens balsamina* | L. | Balsaminaceae | HOSAM407-13 | - | KX783897 |
| 88 | *Impatiens balsamina* | L. | Balsaminaceae | HOSAM406-13 | - | KX783898 |
| 89 | *Ipomoea cairica* | (L.) Sweet | Convolvulaceae | HOSAM113-11 | KX783695 | KX783899 |
| 90 | *Iris pseudacorus* | L. | Iridaceae | HOSAM229-13 | KX783696 | KX783900 |
| 91 | *Jacobaea maritima* | (L.) Pelser & Meijden | Asteraceae | HOSAM283-13 | KX783697 | KX783901 |
| 92 | *Jacobinia suberecta* | André | Acanthaceae | HOSAM368-13 | KX783698 | KX783902 |
| 93 | *Justicia adhatoda* | L. | Acanthaceae | HOSAM289-13 | KX783699 | KX783903 |
| 94 | *Justicia brandegeeana* | Wassh. & L.B.Sm. | Acanthaceae | HOSAM247-13 | KX783700 | KX783904 |
| 95 | *Kalanchoe beharensis* | Drake | Crassulaceae | HOSAM463-13 | - | KX783905 |
| 96 | *Kalanchoe blossfeldiana* | Poelln. | Crassulaceae | HOSAM355-13 | - | KX783906 |
| 97 | *Kalanchoe manginii* | Raym.-Hamet & H.Perrier | Crassulaceae | HOSAM333-13 | - | KX783907 |
| 98 | *Kalanchoe thysiflora* | Balfour | Crassulaceae | HOSAM330-13 | - | KX783908 |
| 99 | *Kalanchoe tomentosa* | Golden Girl | Crassulaceae | HOSAM331-13 | - | KX783909 |
| 100 | *Laccospadix australasica* | L. | Arecaceae | HOSAM351-13 | KX783701 | KX783910 |
| 101 | *Lantana camara* | L. | Verbenaceae | HOSAM362-13 | KX783702 | KX783911 |
| 102 | *Laurus nobilis* | L. | Lauraceae | HOSAM452-13 | KX783703 | KX783912 |
| 103 | *Linum grandiflorum* | L. | Linaceae | HOSAM224-13 | KX783704 | - |
| 104 | *Livistona chinensis* | (Jacq.) R.Br. ex Mart. | Arecaceae | HOSAM449-13 | KX783705 | KX783913 |
| 105 | *Lonicera japonica* | Thunb. | Caprifoliaceae | HOSAM269-13 | KX783706 | KX783914 |
| 106 | *Maranta amagris* | L. | Marantaceae | HOSAM422-13 | KX783707 | KX783915 |
| 107 | *Matricaria chamomilla* | L. | Asteraceae | HOSAM379-13 | KX783708 | KX783916 |
| 108 | *Matthiola incana* | (L.) R.Br. | Brassicaceae | HOSAM193-13 | KX783709 | KX783917 |
| 109 | *Mentha longifolia* | L. | Lamiaceae | HOSAM301-13 | KX783710 | KX783918 |
| 110 | *Mentha longifolia* | L. | Lamiaceae | HOSAM172-11 | KX783711 | KX783919 |
| 111 | *Mentha piperita* | L. | Lamiaceae | HOSAM171-11 | KX783712 | KX783920 |
| 112 | *Mentha piperita* | L. | Lamiaceae | HOSAM302-13 | KX783713 | KX783921 |
| 113 | *Mentha piperita* | Chocolate | Lamiaceae | HOSAM298-13 | KX783714 | KX783922 |
| 114 | *Mentha spicata* | L. | Lamiaceae | HOSAM300-13 | KX783715 | KX783923 |
| 115 | *Mentha spicata* | L. | Lamiaceae | HOSAM173-11 | KX783716 | KX783924 |
| 116 | *Mentha spicata* | L. | Lamiaceae | HOSAM170-11 | KX783717 | KX783925 |
| 117 | *Mentha suaveolens* | Apple mint | Lamiaceae | HOSAM299-13 | KX783718 | KX783926 |
| 118 | *Monstera deliciosa* | Liebm. | Araceae | HOSAM439-13 | KX783719 | KX783927 |
| 119 | *Murraya paniculata* | (L.) Jack | Rutaceae | HOSAM220-13 | KX783720 | KX783928 |
| 120 | *Narcissus jonquilla* | subsp. *jonquilloides* Baker | Amaryllidaceae | HOSAM323-13 | KX783721 | KX783929 |
| 121 | *Narcissus pseudonarcissus* | Ice Follies | Amaryllidaceae | HOSAM321-13 | KX783722 | KX783930 |
| 122 | *Narcissus tazetta* | L. | Amaryllidaceae | HOSAM232-13 | - | KX783931 |
| 123 | *Neoregelia cruenta* | (Graham) L.B.Sm. | Bromeliaceae | HOSAM388-13 | - | KX783932 |
| 124 | *Nephrolepis exaltata* | (L.) Schott | Dryopteridaceae | HOSAM389-13 | - | KX783933 |
| 125 | *Nicotiana alata* | Link & Otto | Solanaceae | HOSAM265-13 | KX783723 | KX783934 |
| 126 | *Olea europaea* | L. | Oleaceae | HOSAM081-10 | KX783724 | KX783935 |
| 127 | *Origanum majorana* | L. | Lamiaceae | HOSAM288-13 | KX783725 | KX783936 |
| 128 | *Oxalis corniculata* | L. | Oxalidaceae | HOSAM214-13 | - | KX783937 |
| 129 | *Pachira glabra* | Pasq. | Malvaceae | HOSAM445-13 | KX783726 | KX783938 |
| 130 | *Pachypodium lamerei* | Drake | Apocynaceae | HOSAM453-13 | KX783727 | KX783939 |
| 131 | *Pandanus veichii* | Mast. | Pandanaceae | HOSAM354-13 | KX783728 | KX783940 |
| 132 | *Papaver rhoeas* | L. | Papaveraceae | HOSAM226-13 | KX783729 | KX783941 |
| 133 | *Parmentiera edulis* | DC. | Bignoniaceae | HOSAM239-13 | KX783730 | KX783942 |
| 134 | *Pelargonium x hortorum* | Patriot Soft Pink | Geraniaceae | HOSAM357-13 | KX783731 | KX783943 |
| 135 | *Pelargonium x hortorum* | Summer Idols Hot Pink | Geraniaceae | HOSAM358-13 | KX783732 | KX783944 |
| 136 | *Pelargonium x hortorum* | 'Patriot Bright Red' | Geraniaceae | HOSAM360-13 | - | KX783945 |
| 137 | *Pelargonium x hortorum* | 'White Rose' | Geraniaceae | HOSAM359-13 | KX783733 | KX783946 |
| 138 | *Pelargonium x hortorum* | Orbit Synchro™ Hot Pink | Geraniaceae | HOSAM361-13 | KX783734 | - |
| 139 | *Pelargonium x hortorum* | Hot Pink | Geraniaceae | HOSAM292-13 | KX783735 | KX783947 |
| 140 | *Pelargonium x hortorum* | Patriot™ Cranberry Red | Geraniaceae | HOSAM274-13 | - | KX783948 |
| 141 | *Pellionia pulchra* | N.E. Br. | Urticaceae | HOSAM237-13 | KX783736 | KX783949 |
| 142 | *Pentas lanceolata* | (Forssk.) Deflers | Rubiaceae | HOSAM261-13 | KX783737 | KX783950 |
| 143 | *Peperomia obtusifolia* | (L.) A.Dietr. | Piperaceae | HOSAM430-13 | KX783738 | KX783951 |
| 144 | *Pericallis malvifolia* | (L'Hér.) B.Nord. | Asteraceae | HOSAM448-13 | KX783739 | KX783952 |
| 145 | *Pericallis x hybrida* | Senetti Blue Bicolor | Asteraceae | HOSAM211-13 | KX783740 | KX783953 |
| 146 | *Pericallis x hybrida* | Senetti Magenta | Asteraceae | HOSAM212-13 | KX783741 | KX783954 |
| 147 | *Pericallis x hybrida* | Senetti Super Blue | Asteraceae | HOSAM210-13 | KX783742 | KX783955 |
| 148 | *Pericallis x hybrida* | Senetti Pink | Asteraceae | HOSAM208-13 | KX783743 | - |
| 149 | *Pericallis x hybrida* | Jester Pure White | Asteraceae | HOSAM209-13 | KX783744 | KX783956 |
| 150 | *Petunia axillaris* | (Lam.) Britton, Sterns & Poggenb. | Solanaceae | HOSAM308-13 | KX783745 | KX783957 |
| 151 | *Petunia integrifolia* | *subsp. inflata* | Solanaceae | HOSAM309-13 | KX783746 | KX783958 |
| 152 | *Petunia x hybrida* | Easy Wave® Yellow | Solanaceae | HOSAM314-13 | KX783747 | KX783959 |
| 153 | *Petunia x hybrida* | Cascadias violet skirt | Solanaceae | HOSAM313-13 | KX783748 | KX783960 |
| 154 | *Petunia x hybrida* | 'Sanguna Picotee Punch' | Solanaceae | HOSAM311-13 | KX783749 | KX783961 |
| 155 | *Petunia x hybrida* | Supertunia® Vista Bubblegum | Solanaceae | HOSAM318-13 | KX783750 | KX783962 |
| 156 | *Petunia x hybrida* | Madness® Rose | Solanaceae | HOSAM310-13 | KX783751 | KX783963 |
| 157 | *Petunia x hybrida* | Surfinia Deep Red | Solanaceae | HOSAM315-13 | KX783752 | KX783964 |
| 158 | *Petunia x hybrida* | Daddy Sugar | Solanaceae | HOSAM312-13 | - | KX783965 |
| 159 | *Philodendron erubescens* | Red Emerald | Araceae | HOSAM202-13 | KX783753 | KX783966 |
| 160 | *Philodendron erubescens* | L. | Araceae | HOSAM438-13 | KX783754 | KX783967 |
| 161 | *Philodendron hederaceum* | (Jacq.) Schott | Araceae | HOSAM440-13 | KX783755 | KX783968 |
| 162 | *Pilea cadierei* | Gagnep. & Guillaumin | Urticaceae | HOSAM395-13 | KX783756 | KX783969 |
| 163 | *Pilea serpyllacea* | (Kunth) Liebm. | Urticaceae | HOSAM416-13 | KX783757 | KX783970 |
| 164 | *Pittosporum tobira* | L. | Pittosporaceae | HOSAM275-13 | KX783758 | KX783971 |
| 165 | *Pittosporum tobira* | Variegata | Pittosporaceae | HOSAM343-13 | KX783759 | KX783972 |
| 166 | *Pittosporum tobira* | L. | Pittosporaceae | HOSAM344-13 | KX783760 | KX783973 |
| 167 | *Plectranthus amboinicus* | Spanish Thyme | Lamiaceae | HOSAM397-13 | KX783761 | KX783974 |
| 168 | *Plectranthus madagascariensis* | Madagascariensis | Lamiaceae | HOSAM415-13 | KX783762 | KX783975 |
| 169 | *Polypodium aureum* | L. | Polypodiaceae | HOSAM249-13 | - | KX783976 |
| 170 | *Polyscias balfouriana* | (André) L.H.Bailey | Araliaceae | HOSAM257-13 | KX783763 | KX783977 |
| 171 | *Ranunculus macranthus* | Scheele | Ranunculaceae | HOSAM200-13 | - | KX783978 |
| 172 | *Ravenea sambiranensis* | Jum. & H.Perrier | Arecaceae | HOSAM444-13 | KX783764 | KX783979 |
| 173 | *Rhaphiolepis indica* | (L.) Lindl. | Rosaceae | HOSAM450-13 | KX783765 | KX783980 |
| 174 | *Rhapis excelsa* | Variegata | Arecaceae | HOSAM418-13 | KX783766 | KX783981 |
| 175 | *Rhipsalis teres* | *f. prismatica* | Cactaceae | HOSAM398-13 | KX783767 | KX783982 |
| 176 | *Rosa hybrida* | Tiffany Pink | Rosaceae | HOSAM277-13 | KX783768 | - |
| 177 | *Rosa hybrida* | Chrysler Imperial Red | Rosaceae | HOSAM278-13 | KX783769 | KX783983 |
| 178 | *Rosmarinus officinalis* | L. | Lamiaceae | HOSAM363-13 | KX783770 | KX783984 |
| 179 | *Rosmarinus officinalis* | L. | Lamiaceae | HOSAM192-13 | KX783771 | KX783985 |
| 180 | *Roystonea regia* | (Kunth) O.F.Cook | Arecaceae | HOSAM474-13 | KX783772 | KX783986 |
| 181 | *Ruellia brittoniana* | Leonard | Acanthaceae | HOSAM404-13 | KX783773 | KX783987 |
| 182 | *Ruellia squarrosa* | (Fenzl) Cufod. | Acanthaceae | HOSAM411-13 | KX783774 | KX783988 |
| 183 | *Ruscus hypoglossum* | L. | Asparagaceae | HOSAM198-13 | KX783775 | -- |
| 184 | *Ruspolia hypocrateriformis* | (Vahl) Milne-Redh. | Acanthaceae | HOSAM447-13 | KX783776 | KX783989 |
| 185 | *Sagina procumbens* | L. | Caryophyllaceae | HOSAM374-13 | - | KX783990 |
| 186 | *Saintpaulia ionantha* | H.Wendl. | Gesneriaceae | HOSAM240-13 | - | KX783991 |
| 187 | *Salvia splendens* | Sellow ex Schult. | Lamiaceae | HOSAM317-13 | KX783777 | - |
| 188 | *Salvia splendens* | Sellow ex Schult. | Lamiaceae | HOSAM259-13 | KX783778 | KX783992 |
| 189 | *Salvia viridis* | L. | Lamiaceae | HOSAM196-13 | KX783779 | KX783993 |
| 190 | *Sansevieria trifasciata* | Prain | Asparagaceae | HOSAM459-13 | KX783780 | KX783994 |
| 191 | *Saxifraga stolonifera* | Curtis | Saxifragaceae | HOSAM238-13 | KX783781 | KX783995 |
| 192 | *Scabiosa atropurpurea* | L. | Caprifoliaceae | HOSAM221-13 | KX783782 | KX783996 |
| 193 | *Sedum burrito* | Moran | Crassulaceae | HOSAM400-13 | - | KX783997 |
| 194 | *Senecio rowleyanus* | H.Jacobsen | Asteraceae | HOSAM399-13 | KX783783 | KX783998 |
| 195 | *Solenostemon scutellarioides* | Brillancy | Lamiaceae | HOSAM381-13 | KX783784 | KX783999 |
| 196 | *Solenostemon scutellarioides* | Chartreuse | Lamiaceae | HOSAM380-13 | KX783785 | KX784000 |
| 197 | *Solenostemon scutellarioides* | Alabama Sunset | Lamiaceae | HOSAM382-13 | KX783786 | KX784001 |
| 198 | *Spathiphyllum wallisii* | Regel | Araceae | HOSAM437-13 | KX783787 | KX784002 |
| 199 | *Spiraea cantoniensis* | Lour. | Rosaceae | HOSAM276-13 | - | KX784003 |
| 200 | *Strelitzia nicolai* | Regel & K.Koch | Strelitziaceae | HOSAM279-13 | KX783788 | KX784004 |
| 201 | *Strelitzia nicolai* | Regel & K.Koch | Strelitziaceae | HOSAM421-13 | KX783789 | KX784005 |
| 202 | *Syngonium podophyllum* | Schott | Araceae | HOSAM155-11 | KX783790 | KX784006 |
| 203 | *Tecomaria capensis* | (Thunb.) Spach | Bignoniaceae | HOSAM219-13 | KX783791 | KX784007 |
| 204 | *Tradescantia spathacea* | Sw. | Commelinaceae | HOSAM393-13 | - | KX784008 |
| 205 | *Tropaeolum majus* | Moonlight | Tropaeolaceae | HOSAM336-13 | KX783792 | KX784009 |
| 206 | *Tropaeolum majus* | Orange Ice | Tropaeolaceae | HOSAM335-13 | KX783793 | KX784010 |
| 207 | *Tropaeolum majus* | Strawberry Ice | Tropaeolaceae | HOSAM337-13 | KX783794 | KX784011 |
| 208 | *Verbena x hybrid* | Groenl. & Rümpler | Verbenaceae | HOSAM334-13 | KX783795 | KX784012 |
| 209 | *Viola tricolor* | Hornveilchen lila | Violaceae | HOSAM370-13 | KX783796 | KX784013 |
| 210 | *Viola tricolor* | Frosthart | Violaceae | HOSAM378-13 | KX783797 | KX784014 |
| 211 | *Viola tricolor* | Hortensis | Violaceae | HOSAM372-13 | KX783798 | KX784015 |
| 212 | *Viola tricolor* | L. | Violaceae | HOSAM377-13 | KX783799 | KX784016 |
| 213 | *Viola tricolor* | Heartsease | Violaceae | HOSAM373-13 | - | KX784017 |
| 214 | *Viola tricolor* | Hornveilchen hellgelb | Violaceae | HOSAM376-13 | KX783800 | KX784018 |
| 215 | *Viola tricolor* | Simon Shine | Violaceae | HOSAM371-13 | KX783801 | KX784019 |
| 216 | *Viola tricolor* | Sun Glory | Violaceae | HOSAM285-13 | KX783802 | KX784020 |
| 217 | *Viola tricolor* | Freefall Purple & White | Violaceae | HOSAM375-13 | KX783803 | KX784021 |
| 218 | *Vitex trifolia* | L. | Lamiaceae | HOSAM294-13 | KX783804 | KX784022 |
| 219 | *Vitis vinifera* | L. | Vitaceae | HOSAM423-13 | KX783805 | KX784023 |
| 220 | *Yucca aloifolia* | purpurea | Asparagaceae | HOSAM349-13 | KX783806 | KX784024 |
| 221 | *Yucca gloriosa* | Variegata | Asparagaceae | HOSAM281-13 | KX783807 | KX784025 |
| 222 | *Yucca gloriosa* | Variegata | Asparagaceae | HOSAM466-13 | KX783808 | KX784026 |
| 223 | *Yucca gloriosa* | Variegata | Asparagaceae | HOSAM346-13 | KX783809 | KX784027 |
| 224 | *Zamioculcas zamiifolia* | (Lodd.) Engl. | Araceae | HOSAM442-13 | KX783810 | KX784028 |
| 225 | *Zantedeschia aethiopica* | (L.) Spreng. | Araceae | HOSAM272-13 | KX783811 | KX783812 |
