# Supplementary material for: The first initiative of DNA barcoding of ornamental plants from Egypt and potential applications in horticulture industry
Source: PLoS One. 2017 Feb 15;12(2):e0172170. doi: 10.1371/journal.pone.0172170 (PMC5310869; doi:10.1371/journal.pone.0172170)
Supplement: S2 Table — % of PCR success based on families is also illustrated. (DOCX) [file pone.0172170.s002.docx]

**S2 Table. Families using each of *matK* & rbcLa of BOLD Taxon ID tree analysis showing that there are 189 sequences, 117 species, 114 genus and 50 family using *matK* and 217 sequences, 131 species, 132 genus and 62 family using *rbcLa*. % of PCR success based on families is also illustrated:**

|  | *matK* | *rbcLa* | *matK rbcL*  *% of PCR success* | |
| --- | --- | --- | --- | --- |
| Family | No. of species | No. of species |  |  |
| Apiaceae | 1 | 1 | 100 | 100 |
| Amaranthaceae | 2 | 2 | 100 | 100 |
| Cactaceae | 1 | 1 | 100 | 100 |
| Caryophyllaceae | - | 1 | 0 | 100 |
| Polygonaceae | 1 | 1 | 100 | 100 |
| Celastraceae | 2 | 2 | 100 | 100 |
| Commelinaceae | 0 | 1 | 0 | 100 |
| Hydrangeaceae | 2 | 2 | 100 | 100 |
| Begoniaceae | 1 | 1 | 100 | 100 |
| Caprifoliaceae | 2 | 2 | 100 | 100 |
| Balsaminaceae | - | 2 | 0 | 100 |
| Fabaceae | 1 | 1 | 100 | 100 |
| Garryaceae | 1 | 1 | 100 | 100 |
| Apocynaceae | 2 | 2 | 100 | 100 |
| Begnoniaceae | 2 | 2 | 100 | 100 |
| Gesneriaceae | - | 1 | 0 | 100 |
| Oleaceae | 1 | 1 | 100 | 100 |
| Plantaginaceae | 2 | 2 | 100 | 100 |
| Lauraceae | 1 | 1 | 100 | 100 |
| Linaceae | 1 | - | 100 | 0 |
| Onagraceae | 1 | 1 | 100 | 100 |
| Oxalidaceae | - | 1 | 0 | 100 |
| Pandanaceae | 1 | 1 | 100 | 100 |
| Piperaceae | 1 | - | 100 | 0 |
| Podocarpaceae | - | 1 | 0 | 100 |
| Cyperaceae | 1` | 1 | 100 | 100 |
| Aspleniaceae | - | 1 | 0 | 100 |
| Dryopteridaceae | - | 1 | 0 | 100 |
| Polypodiaceae | - | 1 | 0 | 100 |
| Papaveraceae | 1 | 1 | 100 | 100 |
| Moraceae | 2 | 2 | 100 | 100 |
| Urticaceae | 3 | 3 | 100 | 100 |
| Rutaceae | 1 | 1 | 100 | 100 |
| Sapindaceae | 1 | 1 | 100 | 100 |
| Saxifragaceae | 1 | 1 | 100 | 100 |
| Convolvulaceae | 2 | 2 | 100 | 100 |
| Vitaceae | 1 | 1 | 100 | 100 |
| Ranunculaceae | 0 | 1 | 0 | 100 |
| Marantaceae | 1 | 1 | 100 | 100 |
| Strelitziaceae | 2 | 2 | 100 | 100 |
| Hydrangeaceae | - | 2 | 0 | 100 |
| Araliaceae | 2 | 2 | 100 | 100 |
| Malvaceae | 4 | 7 | 57.14 | 100 |
| Bromeliaceae | - | 3 | 0 | 100 |
| Asparagaceae | 14 | 14 | 100 | 100 |
| Araceae | 10 | 9 | 100 | 90 |
| Lamiaceae | 23 | 21 | 100 | 91.3 |
| Arecaceae | 11 | 11 | 100 | 100 |
| Asteraceae | 13 | 12 | 100 | 92.30 |
| Brassicaceae | 4 | 6 | 66.66 | 100 |
| Crassulaceae | 1 | 8 | 12.5 | 100 |
| Solanaceae | 11 | 12 | 91.67 | 100 |
| Iridaceae | 4 | 4 | 100 | 100 |
| Euphorbiaceae | 9 | 9 | 100 | 100 |
| Amaryllidaceae | 4 | 5 | 80 | 100 |
| Verbenaceae | 3 | 3 | 100 | 100 |
| Rubiaceae | 3 | 3 | 100 | 100 |
| Acanthaceae | 10 | 10 | 100 | 100 |
| Geraniaceae | 5 | 6 | 83.33 | 100 |
| Pittosporaceae | 3 | 3 | 75% | 100 |
| Rosaceae | 3 | 3 | 100 | 100 |
| Tropaeolaceae | 3 | 3 | 100 | 100 |
| Violaceae | 8 | 9 | 88.89 | 100 |
| Percent of PCR Success based on families |  |  | 75.89 | 96.40 |
