# Supplementary material for: The first initiative of DNA barcoding of ornamental plants from Egypt and potential applications in horticulture industry
Source: PLoS One. 2017 Feb 15;12(2):e0172170. doi: 10.1371/journal.pone.0172170 (PMC5310869; doi:10.1371/journal.pone.0172170)
Supplement: S1 Fig — (DOCX) [file pone.0172170.s006.docx]

S1 Fig. NJ tree of *rbcLa* and *matK* produced in MEGA6 .
