# Supplementary material for: The first initiative of DNA barcoding of ornamental plants from Egypt and potential applications in horticulture industry
Source: PLoS One. 2017 Feb 15;12(2):e0172170. doi: 10.1371/journal.pone.0172170 (PMC5310869; doi:10.1371/journal.pone.0172170)
Supplement: S3 Fig — (DOCX) [file pone.0172170.s008.docx]

**S3 Fig. NJ tree of *rbcL* produced in MEGA6.**
