# Supplementary material for: The first initiative of DNA barcoding of ornamental plants from Egypt and potential applications in horticulture industry
Source: PLoS One. 2017 Feb 15;12(2):e0172170. doi: 10.1371/journal.pone.0172170 (PMC5310869; doi:10.1371/journal.pone.0172170)
Supplement: S1 Sheet — (PDF) [file pone.0172170.s009.pdf]

Glass Fiber Plate DNA Extraction Protocol

Prepared by Natalia Ivanova, Maria Kuzmina and Aron Fazekas

For Plants, Fungi, Echinoderms and Mollusks: *Manual Protocol Employing Centrifugation*

PALL Glass Fiber plate

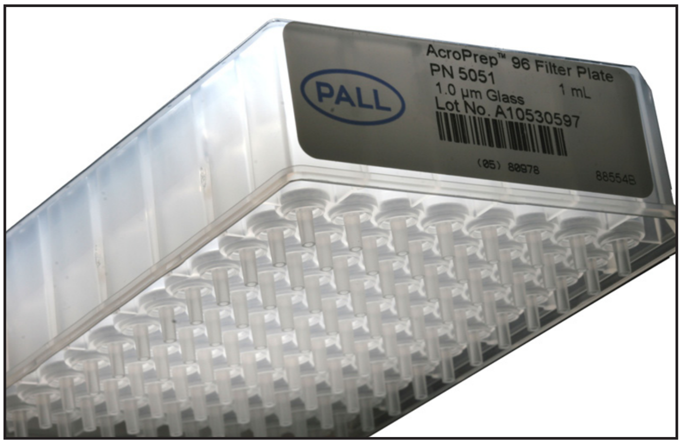

Plant box

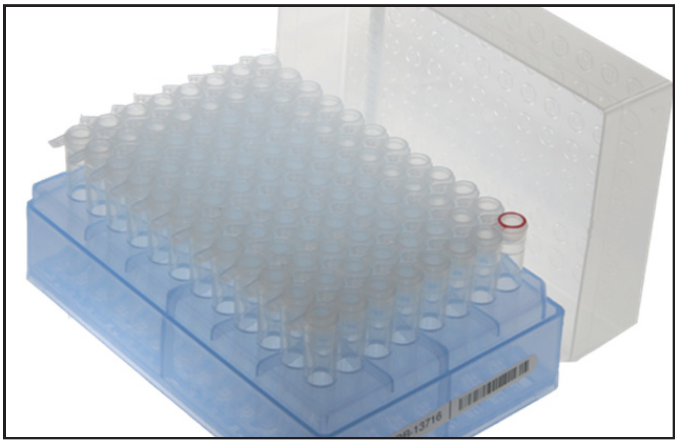

Bind & wash on a square-well block

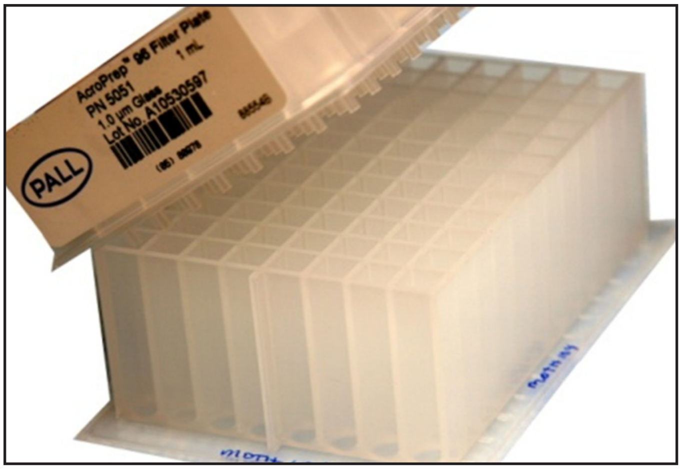

Correct (1-2) and incorrect (3-4) tissue sampling examples for plants

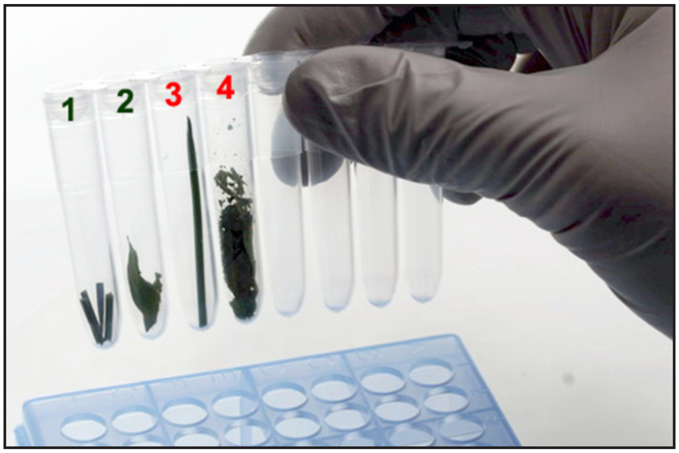

Positioning GF plate for elution step with a PALL collar

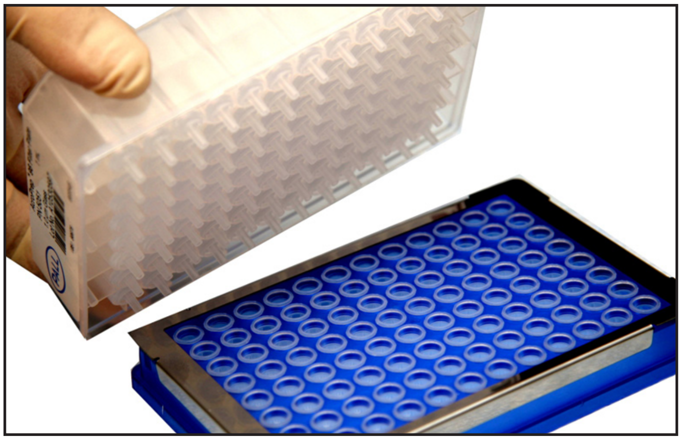

Plant tissue in tube strips with stainless steel beads

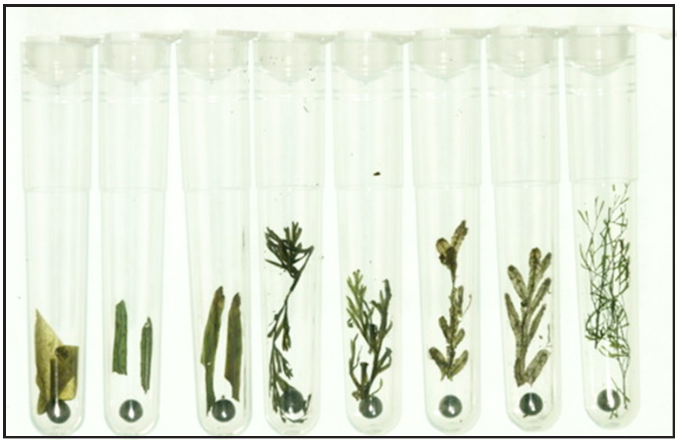

Note: Use 2×CTAB and PALL1 plate for DNA extraction from fungi and plants (alternatively, Insect Lysis Buffer + Na_2SO_3 with Proteinase K can be used for herbarium material). Use 2×CTAB with Proteinase K and PALL2 plate for echinoderms and mollusks. Plant and fungal tissue should be homogenized prior to extraction.

1. Prior to processing, centrifuge plant boxes at 1500 g for 2 min.
2. Add one stainless steel bead to each tube which contains dry tissue (silica gel, dried at 56°C or herbarium sample) and cover with fresh strip caps. Insert boxes, lids removed, into TissueLyser (Qiagen) adapters and shake at 28 Hz for 30 sec, rotate plates and repeat. Centrifuge at 1500 g for 2 min.
3. Open strip caps very carefully by pulling each cap away from the well to avoid cross-contamination by the airborne plant powder. Work on one row of tubes at a time, placing it in separate rack, wipe gloves with ethanol after each row. Add 250-350 µl of 2×CTAB to each tube, cover with fresh strip caps. If working with herbarium material, mix 25 ml of Insect Lysis Buffer + Na_2SO_3 with 2.5 ml of Proteinase K, 20 mg/ml; add 250 µl of mix to each tube, cover with fresh strip caps.
4. Mix once by gentle inverting of fully covered box. Centrifuge at 1500 g for 1 min. Incubate at 65ºC (56ºC – for herbarium material) for 1.5 hours.
5. For mollusks and echinoderms mix 5 ml of 2×CTAB and 0.5 ml of Proteinase K, 20 mg/ml in a sterile container. Add 50 µl of Lysis Mix to each well of 96-well Eppendorf plate containing small pieces of tissue (1-3 mm^3). Cover with fresh strip caps. Incubate at 56 ºC for a minimum of 6 hours or overnight to allow digestion. Centrifuge at 1500 g for 1 min to remove any condensate from the cap strips.
6. For plants, transfer 50 µl of lysate into 96-well Eppendorf plate.
7. Add 100 µl of Plant Binding Buffer (PBB) to each sample using multichannel pipette or Liquidator 96 (Mettler Toledo). Incubate for 5 min at RT.
8. Mix lysate 5-10 times by pipetting, transfer the lysate (about 150 µl) from the wells of microplate into the wells of the GF plate (PALL1 or PALL2) placed on top of a square-well block using multichannel pipette. Seal the plate with self-adhering foil. Centrifuge at 5000 g for 5 min to bind DNA to the GF membrane.
9. First wash step: Add 180 µl of Protein Wash Buffer (PWB) to each well of GF plate. Seal with a new cover and centrifuge at 5000 for 2 min.
10. Second wash step: Add 750 µl of Wash Buffer (WB) to each well of the GF plate. Seal with a new self-adhering foil and centrifuge at 5000 for 5 min.
11. For PALL2 plates only (to avoid incomplete Wash Buffer removal): Open the sealing cover, close it and centrifuge the GF plates again for 5 min at 5000 g.
12. Remove the self-adhering foil. Place GF plate on the lid of a tip box. Incubate at 56 °C for 30 min to evaporate residual ethanol.
13. Position a PALL collar on the collection microplate and place the GF plate on top. Dispense 50 – 60 µl of ddH_20 (prewarmed to 56°C) directly onto the membrane in each well of GF plate and incubate at room temperature for 1 min. Seal plate.
14. Place the assembled plates on a clean square-well block to prevent cracking of the collection plate and centrifuge at 5000 g for 5 min to collect the DNA eluate. Remove the GF plate and discard it.
15. Cover DNA plate with cap strips. DNA can be temporarily stored at 4 ºC or at –20 ºC for long-term storage.
16. Use 1-2 µl of the DNA for PCR.

Additional note: Square-well blocks could be washed with ELIMINase (or with any other DNA removing detergent), autoclaved and re-used. Stainless steel beads can be re-used: separate beads from tissue debris, rinse with water, soak in ELIMINase for 1 hour, wash thoroughly with warm water, soak in 0.5N HCl for 1 min, rinse with warm water followed by dH_2O and final rinse with ddH_2O, dry and expose to UV light for 30 min.

Reagents

Description	Abbreviation	Supplier & Catalogue #
Cetyl trimethylammonium bromide	CTAB	Sigma H9151-250G
Disodium ethylenediamine tetraacetate . $2H_2O$	EDTA	Fisher Scientific S311-500
ELIMINase		Decon Labs Inc. 1102
Ethyl alcohol (anhydrous)	EtOH 96%	Fisher Scientific A962-4
Glycerol	Glycerol	Sigma G5516-500ML
Guanidine thiocyanate	GuSCN	Fisher Scientific BP221-1
Molecular biology grade water	ddH_2O	HyClone SH30538.02
Polyethylene glycol sorbitan monolaurate	Tween-20	MP Biomedicals 194724
Proteinase K	Proteinase K	Invitrogen 25530-031
Sodium chloride	NaCl	Fisher Scientific S271-3
Sodium dodecyl sulfate	SDS	Fisher Scientific BP166-500
Sodium hydroxide	NaOH	Fisher Scientific S318-3
Sodium sulfite	Na_2SO_3	Sigma 239321-500g
t-Octylphenoxypolyethoxy-ethanol	Triton X-100	MP Biomedicals 807426
Tris(hydroxymethyl)amino-metane	Trizma base	Sigma T6066-100g
Tris(hydroxymethyl)amino-metane hydrochloride	Trizma HCl	Sigma T5941-100g

Disposables & Equipment

Description	Abbreviation	Supplier & Catalogue #
ABGene 8-Strip flat PCR caps	cap strips	Fisher AB-0783
AcroPrep 96 1 ml filter plate with 1.0 µm Glass Fiber media, natural housing	PALL1	PALL 5051
AcroPrep 96 1 ml filter plate with 3.0 µm Glass Fiber media over 0.2 µm Bio-Inert membrane, natural housing	PALL2	PALL 5053
Axyseal sealing film	self-adhering foil	Axygene PCR-SP
PROgene Mini Tube System, 1.1 ml	plant box	Progene 24-MTS-11-8-CRS
PROgene 8-strip caps	plant strip caps	Progene 24-MTS-8CP-C
Stainless steel beads, 3.17 mm		Montreal Biotech MBISS44031701
Eppendorf twin.tec 96-well microplates	microplate	Fisher Scientific 361016374
Thermo Scientific Matrix Equalizer Pipette 15-1250 µl, 8-channel	multichannel pipette	Thermo Scientific 2034
Thermo Scientific Matrix 1250 µl, Tall Filter tip (102 mm)	talltips	Thermo Scientific 8245

Stock Solutions

Description	Reagents and Weight		Final Volume
1M Tris-HCl, pH 8.0			
	Trizma base	26.5 g	**500 ml**
	Trizma HCl	44.4 g	
1M Tris-HCl, pH 7.4			
	Trizma base	9.7 g	**500 ml**
	Trizma HCl	66.1 g	
0.1M Tris-HCl, pH 6.4			
	Trizma base	6.06 g	**500 ml**
Note: Adjust pH with HCl to 6.4-6.5.			
1M NaCl			
	NaCl	29.22 g	**500 ml**
0.5 M EDTA pH 8.0			
	EDTA	186.1 g	**1000 ml**
	NaOH	~20.0 g	
Note: Vigorously mix on magnetic stirrer with heater. The disodium salt of EDTA will not go into solution until the pH of the solution is adjusted to ~8.0 by the addition of NaOH. Useful tip – give a brief rinse to NaOH granules with $ddH_2 0$ in a separate glass before dissolving them.			
Proteinase K 20mg/ml, 10 mM Tris-HCl, pH 7.4, 50% glycerol v/v			
	Proteinase K	1000 mg	**50 ml**
Note: Add 0.5 ml of 1 M Tris pH 7.4 and 10-15 ml $ddH_2 0$ to a 1000 mg package of Proteinase K; dissolve, pour into graduated cylinder, adjust volume to 25 ml with $ddH_2 0$, add 25 ml of glycerol; mix, aliquot by 1 ml. Store at – 20°C (glycerol prevents freezing and protects enzyme).			

Additional note: Thoroughly wash labware with ELIMINase, rinse with $dH_2 0$. Weigh reagents using a clean spatula, fill up with the molecular grade $ddH_2 0$ to the final volume. Filter buffers through 0.2 µm filter into a clean bottle; make smaller volume working aliquots (e.g. 100 ml). Store stock solutions and working aliquots at 4°C.

Working Solutions for DNA Extraction

Description	Reagents and Volume (ml)/ Weight (g)		Final Volume
2×CTAB			
2%CTAB	CTAB	4.0 g	**200 ml**
100 mM Tris-HCl, pH 8.0	1M Tris-HCl, pH 8.0	20 ml	
20 mM EDTA, pH 8.0	0.5M EDTA, pH.8.0	8 ml	
1.4 M NaCl	NaCl	16.4 g	
Note: Dissolve first three components in 150 ml of ddH$_2$0, then add NaCl, mix on magnetic stirrer with heater.			
Insect Lysis Buffer+Na_2SO_3			
700 mM GuSCN	GuSCN	16.5 g	**200 ml**
30 mM EDTA pH 8.0	0.5M EDTA, pH.8.0	12 ml	
30 mM Tris-HCl pH 8.0	1M Tris-HCl, pH 8.0	6 ml	
0.5% Triton X-100	Triton X-100	1 ml	
5% Tween-20	Tween-20	10 ml	
52 mM Na_2SO_3			
Note: Dissolve first five components in 200 ml of ddH$_2$0, vigorously mix on magnetic stirrer with heater. Add Na_2SO_3 just prior to use.			
Binding Buffer (BB) - stock			
6M GuSCN	GuSCN	354.6 g	**500 ml**
20 mM EDTA pH 8.0	0.5M EDTA, pH.8.0	20 ml	
10 mM Tris-HCl pH 6.4	0.1M Tris-HCl, pH 6.4	50 ml	
4% Triton X-100	Triton X-100	20 ml	
Note: Vigorously mix on magnetic stirrer with heater. If any re-crystallization occurs, pre-warm at 56ºC to dissolve before use.			
Plant Binding Buffer (PBB)			
	Binding Buffer	80 ml	**96 ml**
	ddH$_2$0	16 ml	
Protein Wash Buffer (PWB)			
	Binding Buffer	50 ml	**100 ml**
	EtOH 96%	50 ml	
Note: stable at room temperature for 1 month.			
Wash Buffer (WB)			
60 % EtOH	EtOH 96%	300 ml	**475 ml**
50 mM NaCl	1M NaCl	23.75 ml	
10 mM Tris-HCl, pH 7.4	1M Tris-HCl, pH 7.4	4.75 ml	
0.5 mM EDTA, pH 8.0	0.5M EDTA, pH 8.0	0.475 ml	
Note: mix well, store at –20ºC.			

Additional note: Weigh the dry components (e.g. SDS or GuSCN) first, then add required volumes of the stock solutions, and fill up with the molecular grade ddH$_2$0 to the final volume. No filtering is required.

References

Ivanova NV, deWaard JR, Hebert PDN (2006) An inexpensive, automation-friendly protocol for recovering high-quality DNA. Molecular Ecology Notes 6, 998-1002.

Ivanova NV, Fazekas AJ, Hebert PDN (2008) Semi-automated, membrane-based protocol for DNA isolation from plants. Plant Molecular Biology Reporter 26, 186-198.

Whitlock R, Hipperson H, Mannarelli M, Burke T (2008) A high-throughput protocol for extracting high-purity genomic DNA from plants and animals. Molecular Ecology Resources 8, 736-741.
