# Supplementary material for: The first initiative of DNA barcoding of ornamental plants from Egypt and potential applications in horticulture industry
Source: PLoS One. 2017 Feb 15;12(2):e0172170. doi: 10.1371/journal.pone.0172170 (PMC5310869; doi:10.1371/journal.pone.0172170)
Supplement: S2 Sheet — (PDF) [file pone.0172170.s010.pdf]

PCR Amplification for Plants and Fungi

Prepared by Maria Kuzmina and Natalia Ivanova

Taq Polymerase Choice

High DNA barcoding production rates demand high success in amplification of the barcode region. One particularly critical element for PCR amplification is the polymerase enzyme. During the testing of DNA barcoding protocols across a broad range of taxonomic groups, from animals to plants, it was clear that one higher-cost polymerase from Invitrogen (Platinum® Taq DNA Polymerase) delivered both greater-intensity amplicons and amplification success in cases where standard Taq failed. Results indicated that Platinum® Taq offers the highest performance, and it is now the standard PCR enzyme used by the CCDB. Platinum® Taq also offers a number of benefits over standard Taq polymerases. It is a robust enzyme that needs less optimization compared to standard Taq. As Platinum® Taq requires a "hot start" for activation, there is less enzyme breakdown and fewer non-specific PCR amplicons. Platinum® Taq is also stable at room temperature, allowing for advanced preparation and storage of PCR plates for future use.

Addition of trehalose facilitates PCR and makes possible freezing of aliquoted master-mixes. Currently CCDB uses batch strategy for making PCR plates. Mixes are aliquoted directly into 96-well plates; plates are covered with PCR film and stored at -20°C for up to 3 months. Each batch is labeled, recorded in the system and tested to assure performance. The combination of a thermostable Platinum® Taq with trehalose ensures high performance even after multiple freeze-thaws. Results with regular Taq polymerases may be less satisfactory.

Aliquots in tubes can be stored at -20°C for up to 3 months (1-3 freeze-thaw cycles don't affect performance). The contents of a tube should be mixed by pipetting before use.

Amplification of different plant markers requires different conditions for PCR reaction (refer to tables below). Phusion® Hot Start High-Fidelity DNA Polymerase has proved to be very efficient in amplification of homopolymer regions in psbA-trnH and greatly improves sequencing results.

Consumables & Equipment for PCR amplification

- 10% trehalose: dissolve 5 g D-(+)-trehalose dehydrate, in 50 ml of molecular grade ddH$_2$O; or for 20% dissolve 10 g of trehalose in 50 ml of molecular grade ddH$_2$O.
- 10X PCR Buffer for Platinum Taq (Invitrogen). Store at –20°C.
- 50 mM MgCl$_2$ (Invitrogen).Store at –20°C.
- 10 mM dNTP mix (Kapa Biosystems). Store at –20°C in 100 µl aliquots.
- 100 µM primer stock: dissolve desiccated primer (Invitrogen) in – number of nmol x 10 µl ultrapure H$_2$O. Store at –20°C.
- 10 µM primer working solution: add 20 µl 100 µM primer stock to 180 µl of molecular grade ddH$_2$O. Store at –20°C.
- Platinum Taq polymerase (Invitrogen) or Phusion Hot Start High-Fidelity DNA Polymerase (Finnzymes). Store at –20°C in 50 µl aliquots.
- Microplate (Eppendorf).
- Cap strips (ABgene) or heat-sealing film.
- Thermocycler (Mastercycler EP Gradient, Eppendorf).

Basic Recipe for PCR for rbcL and ITS2 Markers

PCR reagents per 12.5 µl reaction:

# of reactions	1	100
10% trehalose	6.25 µl	625 µl
ddH$_2$0	2 µl	200 µl
10X buffer	1.25 µl	125 µl
50 mM MgCl$_2$	0.625 µl	62.5 µl
10 µM primer A	0.125 µl	12.5 µl
10 µM primer B	0.125 µl	12.5 µl
10 mM dNTPs	0.0625 µl	6.25 µl
Polymerase (5 U/µl)	0.06 µl	6 µl
**Total**	**10.5 µl**	**1050 µl**
DNA template	2 µl per well	

Aliquot 1/8 of total mix volume in 8-tube PCR strip (if making more than one plate – to disposable container) and dispense desired volume (10.5 µl for 12.5 µl reactions) in 96-well plate and then add 1-2 µl of DNA extract (30-50 ng/µl). If you plan to fill several 96-well plates include extra volume to allow for pipetting mistakes and dead volume in the digital multichannel pipettor (e.g. for making 10 plates with 12.5 µl reactions each, include about 40 extra reactions).

Basic Recipe for PCR for matK Marker

PCR reagents per 6.5 µl reaction:

# of reactions	1	100
20% trehalose	1.875 µl	187.5 µl
ddH$_2$0	2.6 µl	260 µl
10X buffer	0.75 µl	75 µl
50 mM MgCl$_2$	0.225 µl	22.5 µl
10 µM primer A	0.375 µl	37.5 µl
10 µM primer B	0.375 µl	37.5 µl
10 mM dNTPs	0.15 µl	15 µl
Polymerase (5 U/µl)	0.15 µl	15 µl
**Total**	**6.5 µl**	**650 µl**
DNA template (diluted 10x)	1 µl per well	

Aliquot 1/8 of total mix volume in 8-tube PCR strip (if making more than one plate – to disposable container) and dispense desired volume (6.5 µl for 7.5 µl reactions) in 96-well plate and then add 1-2 µl of 10x diluted DNA extract (3-5 ng/µl). If you plan to fill several 96-well plates include extra volume to allow for pipetting mistakes and dead volume in the digital multichannel pipettor (e.g. for making 10 plates with 6.5 µl reactions each, include about 40 extra reactions). However, we do not recommend long term storage of matK pre-made plates due to a small reaction volume.

Basic Recipe for PCR for psbA-trnH Marker

PCR reagents per 10 µl reaction:

# of reactions	1	100
DMSO	0.3 µl	30 µl
ddH_20	6.32 µl	632 µl
5X buffer HF (with $MgCl_2$)	2 µl	200 µl
10 µM primer A	0.1 µl	10 µl
10 µM primer B	0.1 µl	10 µl
10 mM dNTPs	0.056 µl	5.6 µl
Phusion Hot-Start (5 U/µl)	0.125 µl	12.5 µl
**Total**	**9 µl**	**900 µl**
DNA template	1 µl per well	

Aliquot 1/8 of total mix volume in 8-tube PCR strip (if making more than one plate – to disposable container) and dispense desired volume (9 µl for 10 µl reactions) in 96-well plate and then add 1 µl of DNA extract (30-50 ng/µl). If you plan to fill several 96-well plates include extra volume to allow for pipetting mistakes and dead volume in the digital multichannel pipettor (e.g. for making 10 plates with 10 µl reactions each, include about 40 extra reactions). These plates could not be stored at -20°C.

General Recommendations

- The use of filter tips is recommended for all PCR reagents to avoid contamination. Clean the bench top with alcohol before setting up reactions. Always use a sterile tip when removing Taq polymerase and the other reagents from their tubes.
- Keep DNA templates (i.e. other PCR products) away from the PCR reagents while you are setting up the reaction mixes. Add DNA after all of the reagents have been returned to the freezer.
- Always include a sample without template as a negative control to check for contamination of the reagents. Include a positive control (a DNA sample that has amplified in the past) as well to test the effectiveness of the PCR reagents.

Tips for Primer Design

- Primers should be between 20 - 30 nt in length.
- Avoid complementarity within and between primers.
- The GC content should be approximately 50%.
- Avoid mono- or dinucleotide repetition within primers.
- The primer should end on a G or a C.
- Primers should end on the second (or the first if necessary) position of a codon.
- The melting temperatures of primer pairs should be within 5°C.
- To design primers for a particular taxonomic group, try aligning as many genes from closely related taxa as possible (try surfing GenBank) for the desired species group. Design primers that are situated in regions that are conserved across all taxa.
- Primers could be tailed with M13 tails. However, some tailed versions can form strong primer dimers, reducing PCR efficiency.

PCR Thermocycle Programs

rbcL: 94°C for 4 min; 35 cycles of 94°C for 30 sec, 55°C for 30 sec, 72°C for 1 min; final extension 72°C for 10 min.

trnH–psbA using Phusion polymerase: 98°C for 45 sec; 35 cycles of 98°C for 10 sec, 64°C for 30 sec, 72°C for 40 s; final extension 72°C for 10 min.

matK first round (matK-KIM1R-f/matK-KIM3F-r): 94°C for 1 min; 35 cycles of 94°C for 30 sec, 52°C for 20 sec, 72°C for 50 sec; final extension 72°C for 5 min.

matK second round failure-tracking (matK-390f/matK-1326r): 94°C for 1 min; 35 cycles of 94°C for 30 sec, 50°C for 40 sec, 72°C for 40 sec; final extension 72°C for 5 min.

ITS2 (ITS2-S2F/ITS4): 94°C for 5 min; 35 cycles of 94°C for 30 sec, 56°C for 30 sec, 72°C for 45 sec; final extension 72°C for 10 min.

PCR Product Check

Invitrogen E-gel® 96 system and software

We now employ pre-cast agarose gels from Invitrogen. This system is bufferless, so exposure to Ethidium Bromide is minimized. However, gloves should be worn when handling and loading the gel.

Loading and running E-gel® 96 gels

The recommended program for 2% Agarose E-gel® 96 gel is EG and the run time is 4-6 min. Plug the Mother E-Base into an electrical outlet. Press and release the pwg/prg (power/program) button on the base to select program EG.

- Remove gel from the package; remove plastic comb from gel.
- Slide gel into the two electrode connections on the Mother or Daughter E-Base™.
- Load 12-16 µl of ddH_20 into wells with 8- or 12-multichannel pipettor.
- Load appropriate DNA markers in the marker wells.
- Load 4 µl of sample.
- To begin electrophoresis, press and release the pwd/prg button on the E-Base™. The red light changes to green.
- At the end of run (signaled with a flashing red light and rapid beeping), press/release the pwr/prg button to stop the beeping.
- Remove gel cassette from the base and capture a digital image of a gel on UV transilluminator equipped with digital camera.
- Analyze the image and align or arrange lanes in the image using the E-editor™2.0 software available at http://www.invitrogen.com/egels
- Incorporate E-gel image into lab spreadsheet for estimation of concentration and hit picking.

PCR product clean-up

Currently we don't clean-up PCR products and proceed

directly to sequencing.

Typical E-gel image

Plant DNA was extracted with Glass Fiber method (refer to DNA extraction section) and amplified with Platinum Taq polymerase using rbcLa-F and rbcLa-R primers (master mix contained 5% trehalose)

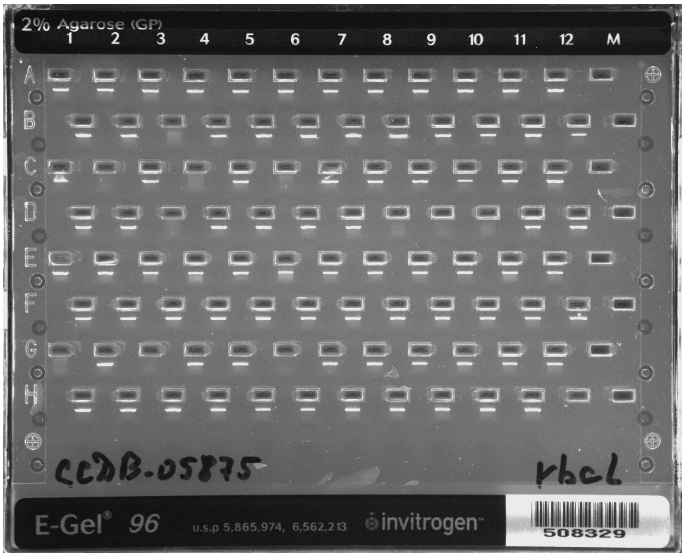

The white bands indicate products; the square slots are the loading wells; H12 – negative control

References

CBOL Plant Working Group (2009) A DNA Barcode for land plants. PNAS, 106 (31): 12794-12797.

Fazekas A, Steeves R, Newmaster S (2010) Improving sequencing quality from PCR products containing long mononucleotide repeats. Biotechniques 48:277-285.

Franks F (1990) Freeze drying: from empiricism to predictability. Cryoletters 11:93-110.

Hajibabaei M, deWaard JR, Ivanova NV et al. (2005) Critical factors for assembling a high volume of DNA barcodes. Philosophical Transactions of the Royal Society: Biological Sciences 360:1959 – 1967.

Ivanova N, Grainger C (2006) Pre-made frozen PCR and sequencing plates. CCDB Advances, Methods Release No. 4, December 1st, 2006.

Ivanova N, Grainger C, Hajibabaei M (2006) Increased DNA barcode recovery using Platinum® Taq. CCDB Advances, Methods Release No 2, November 3, 2006.

Ivanova NV, deWaard JR, Hajibabaei M, Hebert PDN (2005) Protocols for high volume DNA barcoding. Draft submission to: DNA working group Consortium for the Barcode of Life. Published online at http://www.dnabarcoding.ca/

Spiess AN, Mueller N, Ivell R (2004) Trehalose is a potent PCR enhancer: Lowering of DNA melting temperature and thermal stabilization of Taq polymerase by the disaccharide trehalose. Clinical Chemistry 50:1256-1259.
